# Colorimetric fluoride detection in dimethyl sulfoxide using a heteroleptic ruthenium(ii) complex with amino and amide groups: X-ray crystallographic and spectroscopic analyses†

**DOI:** 10.1039/d2ra03593f

**Published:** 2022-09-07

**Authors:** Mari Toyama, Tomoki Hasegawa, Noriharu Nagao

**Affiliations:** Department of Engineering Science, Faculty of Engineering, Osaka Electro-Communication University 18-8 Hatsucho Neyagawa Osaka 572-8530 Japan m-toyama@osakac.ac.jp; Department of Chemistry of Functional Molecules, Faculty of Science and Engineering, Konan University 8-9-1 Okamoto, Higashinada Kobe Hyogo 658-8501 Japan; Department of Applied Chemistry, School of Science and Technology, Meiji University 1-1-1 Higashimita, Tama Kawasaki Kanagawa 214-8571 Japan

## Abstract

A bis-heteroleptic ruthenium(ii) complex, [Ru(Hdpa)_2_(H_2_pia)]X_2_ (1·X_2_; X = Cl, OTf, or F; Hdpa = di-2-pyridylamine; H_2_pia = 2-pycolinamide; OTf^−^ = CF_3_SO_3_^−^), was synthesized and spectroscopically and crystallographically characterized. The crystal structures of 1·Cl_2_·2.5H_2_O and 1·F_2_·2EtOH revealed essentially identical geometries for the 1^2+^ dication; however, the dihedral angle between the two pyridyl groups in the Hdpa ligands, which represented the degree of bending of the bent conformation, was affected by hydrogen-bonding interactions between the NH group and counterions. In 1·F_2_·2EtOH, one of the Hdpa ligands had an unusually smaller dihedral angle (15.8°) than the others (29.9°–35.0°). The two NH groups of each Hdpa ligand and the NH_2_ group of the H_2_pia ligand in 1^2+^ acted as receptors for F^−^ anion recognition *via* hydrogen-bonding interactions in a dimethyl sulfoxide (DMSO) solution, and the reaction showed an unambiguous color change in the visible region. Upon the addition of tetra-*n*-butylammonium fluoride to the red DMSO solution of 1·(OTf)_2_·H_2_O, the solution turned dark brown. ^1^H NMR analysis and absorption spectroscopy of the reaction between 1^2+^ and the added F^−^ anions revealed that the F^−^ anions did not distinguish between the two amino groups of Hdpa and the amide group of H_2_pia, although they were in different environments in the DMSO solution. A tris-F-adduct with 1^2+^, 1·F_3_^−^, was formed when sufficient F^−^ anions were present in the solution, despite the presence of four NH protons in 1^2+^. Time-dependent DFT calculations of 1^2+^ and 1·F_3_^−^ were consistent with their absorption spectra.

## Abstract 2

A bis-heteroleptic ruthenium(ii) complex; [Ru(Hdpa)_2_(H_2_pia)]X_2_ (1·X_2_; X = Cl, OTf, or F; Hdpa = di-2-pyridylamine; H_2_pia = 2-pycolinamide; OTf^–^ = CF_3_SO_3_^–^) was synthesized and spectroscopically and crystallographically characterized.

## Graphical abstract

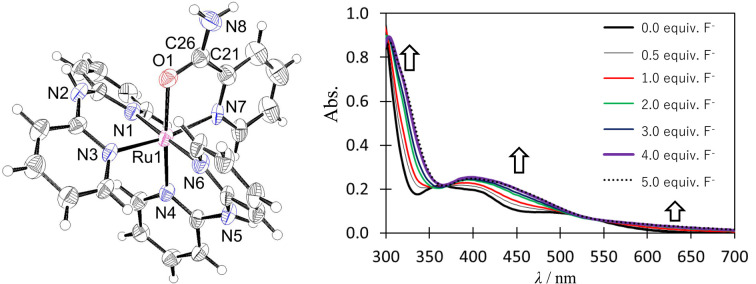

## 1 Introduction

Recognition of toxic anions has recently become a significant aspect of supramolecular and coordination chemistry owing to its potential applications in environmental, biological, industrial, and agricultural fields.^1–11^ In particular, fluoride (F^−^) sensing is attracting considerable attention because of the small size, high charge density, and hard Lewis characteristics of F^−^. Fluoride is used to treat osteoporosis and maintain dental health, and is readily absorbed by the human body but excreted sluggishly. Excessive ingestion of F^−^ can cause fluorosis, nephrotoxic changes, and urolithiasis in humans.^12–14^ Compounds containing NH protons, such as imidazoles, pyrazoles, amides, amino, thiourea, and urea derivatives, have been extensively used as chromogenic and fluorogenic sensors for anions, and naturally for F^−^ anions.^1,2,13–25^ This is due to the ability of the NH proton to participate in strong hydrogen-bonding interactions with F^−^.^16^

Transition metal complexes have been used for signal modification and absorption- (colorimetric) or emission-based analyses owing to their receptor–anion interactions. Several ruthenium(ii) complexes derived from polypyridyl ligands such as imidazole, pyrazole, amide, amino, hydroxy, and urea moieties have been designed and investigated as anion sensors.^16,23,26–28^ Most research on anion sensing has targeted absorption or emission spectroscopy. Moreover, ‘naked-eye’ colorimetric and fluorometric detection has been achieved.^14,18,22^ These sensing features are noteworthy because compounds or complexes can be used as qualitative anion agents without analytical equipment. Patil *et al.* reported the reactions of three types of Ru(ii)–Hdpa complexes, [Ru(bpy)_*n*_(Hdpa)_3−*n*_]^2+^ (bpy = 2,2′-bipyridyne, Hdpa = di-2-pyridylamine; *n* = 0–2), with nine anions (F^−^, Cl^−^, Br^−^, PF_6_^−^, NO_3_^−^, ClO_4_^−^, HSO_4_^−^, CH_3_COO^−^, and CN^−^).^22^ The Hdpa ligands in the complex were found to act as selective colorimetric sensors for F^−^ and CN^−^ anions with a detectable color change in the visible region. Moreover, the stoichiometries of their Ru(ii)–Hdpa complexes for F^−^ or CN^−^ anions were 1 : 1, 1 : 2, and 1 : 3, depending on the number of Hdpa ligands in the complex.

**Scheme 1:**
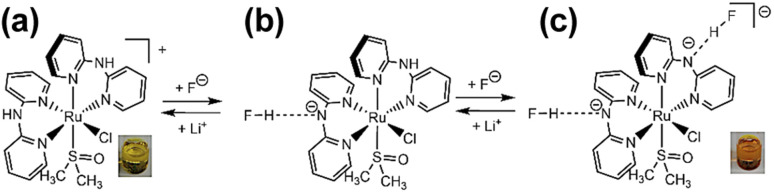
Two NH groups of each Hdpa ligands in *cis*-[RuCl(dmso-*S*)(Hdpa)_2_]^+^ [structure (a)] reacted with added F^−^ anions to form *cis*-[RuCl(dmso-*S*)(FHdpa)_2_]^−^ [structure (c)] *via cis*-[RuCl(dmso-*S*)(FHdpa)(Hdpa)] [structure (b)]. The solution of the original complex was yellow, and after adding the F^−^ anions, it turned orange. Li^+^ was added to the orange solution and it returned to the original yellow color recovering *cis*-[RuCl(dmso-*S*)(Hdpa)_2_]^+^ [structure (a)] and LiF.

**Scheme 2:**
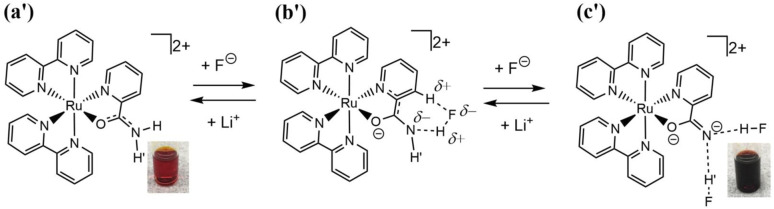
Two amide protons of the H_2_pia ligand in [Ru(bpy)_2_(H_2_pia)]^2+^ [structure (a′)] reacted with the added F^−^ anions to form [Ru(bpy)_2_(F_2_H_2_pia)] [structure (c′)] *via* [Ru(bpy)_2_(FH_2_pia)]^+^ [structure (b′)]. The reaction of the original complex was red, and after adding F^−^ anions, it turned dark red. Li^+^ was added to the dark red solution, and it returned to the original red color, recovering [Ru(bpy)_2_(H_2_pia)]^2+^ [structure (a′)] and LiF.

Two types of polypyridyl ruthenium(ii) complexes, *cis*-[RuCl(dmso-*S*)(Hdpa)_2_](OTf) (P1·(OTf); dmso = dimethyl sulfoxide and OTf^−^ (triflate) = CF_3_SO_3_^−^) and [Ru(bpy)_2_(H_2_pia)](OTf)_2_ (P2·(OTf)_2_; H_2_pia = 2-picolineamide) were synthesized in our previous study.^29,30^ These Ru(ii) complexes, which have amino (NH) or amide (NH_2_CO) groups in the polypyridyl ligands, function as F^−^ sensors. The reactions of these complexes with F^−^ anions are illustrated in Schemes 1 and 2. The addition of the Ru(ii)–Hdpa complex, which has an amino group in each Hdpa ligand, to an acetonitrile or DMSO solution for reacting with F^−^ anions results in the yellow solution turning orange (Scheme 1). ^1^H NMR analysis of the reaction between the bis-Hdpa–Ru(ii) complex [Scheme 1; structure (a)] and F^−^ anions suggested that the added F^−^ anions could distinguish the two Hdpa ligands. The first F^−^ reacted with the Hdpa ligand in *trans* orientation with respect to the Cl ligand to form *cis*-[RuCl(dmso-*S*)(FHdpa)(Hdpa)] [Scheme 1; structure (b)], and then, the second F^−^ reacted with another Hdpa ligand in *trans* orientation with respect to the dmso-*S* ligand to yield *cis*-[RuCl(dmso-*S*)(FHdpa)_2_]^−^ [Scheme 1; structure (c)]; thus, two-step reactions were observed to form mono- and bis-F-adduct complexes.^29^ In the case of the Ru(ii)–H_2_pia complex, crystallographic analysis revealed that the H_2_pia ligand was coordinated to a Ru^2+^ ion *via* pyridyl-N and amide-O atoms [Scheme 2; structure (a′)]. When fluoride anions are added to an acetonitrile solution of the Ru(ii)–H_2_pia complex, the red solution turns deep red. This is due to the transformation of the coordinated neutral O atom into slightly negative O^−^ as well as the interactions of F^−^ with the amide protons [Scheme 2; structure (b′)], which lead to unambiguous changes in the metal-to-ligand charge-transfer (MLCT) band that enable the complex to achieve naked-eye detection of the fluoride anion. Spectrophotometric titration indicated that the two amide protons reacted with the added F^−^ anions in a two-step manner. ^1^H NMR spectra suggested that, in the first step of the reaction, the F^−^ anion formed two hydrogen bonds with an amide proton and the H-3 proton of the pyridyl group in the H_2_pia ligand to form a seven-membered chelate ring. This fluoride-containing chelating structure, with two hydrogen bonds, an NH group, and the H-3 proton of an aromatic ring or a related CH component, has also been proposed for some organic compounds (Fig. S1†).^16,17^ After the first step, a second F^−^ anion interacts with the remaining amide proton, and the link between the first F^−^ anion and H-3 proton is severed. [Scheme 2; structure (c′)] This indicates that the reaction of a Ru(ii)–H_2_pia complex with F^−^ anions can be characterized by monitoring the chemical shifts of the H-3 signal of the H_2_pia ligand during titration with F^−^ anions.^30^ Moreover, ^1^H NMR analysis of the reaction of both complexes with F^−^ anions indicated that F^−^ forms stronger links with an NH proton or amide protons compared to a Cl^−^ anion based on the observed shifts of all signals of the pyridyl group, especially the H-5 signal, in the Hdpa ligand. Furthermore, a suitable amount of Li(OTf) was added to the F-adduct complex solution in both scenarios, and an original complex with free F^−^ anions was recovered to yield LiF.^29,30^

In 2021, Gong and Zhong reported the structure of a bis-heteroleptic complex [Ru(bpy)_2_(Hpia^−^)](PF_6_), which had two types of chelate ligands, and its reaction with F^−^, in which Hpia^−^, a deprotonated form of H_2_pia, was coordinated to the Ru^2+^ ion *via* pyridyl-N and amide-N to act as an anionic *N*,*N*′ bidentate ligand.^11^ Interestingly, no distinct absorption spectral changes were observed for the complex in the presence of the F^−^ anion, although the complex had an NH group in Hpia^−^. Their results revealed that the coordination form of the Ru(ii)–H_2_pia complex, in which H_2_pia had the *N*,*O*-coordination mode, was important for naked-eye fluoride detection.

The synthesis, crystal structures, and reactivity of a new bis-heteroleptic complex [Ru(Hdpa)_2_(H_2_pia)]X_2_ (1·X_2_; X = Cl, OTf, or F), which was obtained by reacting P1^+^ with an H_2_pia ligand, are described herein. The bis-heteroleptic complex 1^2+^ has two different types of F^−^ sensing sites: two NH groups in the two Hdpa ligands and two amide protons in the H_2_pia ligand. The investigation was performed to determine the possibilities of all four protons in 1^2+^—one amide and two amino groups—interacting with F^−^ anions, and to ascertain the existence of a relationship between its Hdpa and H_2_pia ligands for reacting with F^−^ anions. Ru(ii)–Hdpa and Ru(ii)–H_2_pia complexes have previously been found to be monocationic and dicationic, respectively. Therefore, a comparison between the reactivities of coordinated Hdpa and H_2_pia ligands in Ru(ii) ion complexes for F^−^ anions has not been sufficiently performed. The heteroleptic complex 1^2+^, which has two types of bidentate ligands—Hdpa and H_2_pia—enables direct comparison of their reactivities and relationship with respect to fluoride. However, signals of amino and amide protons were not observed in the ^1^H NMR spectra of the Ru(ii)–Hdpa and Ru(ii)–H_2_pia complexes in D_2_O and CD_3_OD, indicating that these protons were quickly exchanged by those of solvent molecules. Therefore, the colorimetric fluoride detection strategy based on the Hdpa- or H_2_pia-ligand-containing complex was ineffective in water and alcohols. The hydrogen-bonding interactions between F^−^ anions and the Hdpa or H_2_pia in the Ru(ii) complexes were investigated exclusively in organic solutions—DMSO and acetonitrile. However, the reaction of 1^2+^ with F^−^ in DMSO was particularly targeted because 1·F_2_ was poorly soluble in acetonitrile.

## 2 Experimental

### 2.1 General

The starting complex, *cis*-[RuCl(dmso-*S*)(Hdpa)_2_]Cl (P1·Cl), was prepared according to a previously reported method.^29^ All reactions were performed in an argon atmosphere. ^1^H NMR spectroscopy and ^1^H–^1^H correlation spectroscopy (COSY) (Fig. S2–S4†) were performed using DMSO-*d*_6_ and CD_3_CN at 298 K with Varian Mercury 300 (300 MHz) or Varian UNITY INOVA 500 (500 MHz) spectrometers. Tetramethylsilane (TMS) was used as the internal standard for DMSO-*d*_6_ and CD_3_CN. Absorption spectra were recorded at approximately 298 K using a HITACHI U-3310 spectrometer with DMSO.

X-ray crystallographic analysis of [Ru(Hdpa)_2_(H_2_pia)]Cl_2_·2.5H_2_O (1·Cl_2_·2.5H_2_O) was performed at 173 K using a Rigaku XtaLAB P200 diffractometer with multilayer-mirror-monochromated Cu Kα radiation (*λ* = 1.5418 Å) and *θ* values of 4.355°–68.237°, whereas that of [Ru(Hdpa)_2_(H_2_pia)]F_2_·2EtOH (1·F_2_·2EtOH) was performed at 173 K using a Rigaku Mercury70 diffractometer with graphite monochromated Mo Kα radiation (*λ* = 0.71073 Å) and *θ* values of 2.11°–30.35°. The structure was solved using the direct method with the SIR2011 and SIR2014 packages and refined using full-matrix least-squares techniques.^31–34^ All non-hydrogen atoms were refined with anisotropic displacement parameters. The hydrogen atoms of the pyridyl rings and amino groups were located at positions theoretically calculated using the riding model. For 1·Cl_2_·2.5H_2_O, the hydrogen atoms from the amide group of H_2_pia in the complex were assigned based on a difference Fourier map. *U*_iso_(H) was set to 1.2*U*_eq_(C,N) for H atoms. During the refinement, a satisfactory result (*R*1 = 0.0875) was obtained by modeling the disordered O atoms of 2H_2_O (solvent: water); however, 0.5H_2_O was missing from the composition determined by elemental analysis. Thus, the SQUEEZE routine^34^ in the *PLATON* program^35^ was used to generate a modified dataset in which the contribution of the disordered solvent molecules to the structure amplitudes was discarded. Voids with volumes of 176 and 141 Å^3^ at (0, 0.5, 1) and (0.492, 1, 0.5), which were occupied by the disordered solvent (20.3% of the unit-cell volume), contained 41 and 35 electrons, respectively. Although the estimated 76 electrons were fewer than the 90 electrons expected for five H_2_O molecules per unit cell, three and two H_2_O molecules were assumed to be present in the former and latter voids, respectively, based on the elemental analysis of 1·Cl_2_·2.5H_2_O. The solvent molecules are included in the reported molecular formula as well as the weight and density calculations.

For 1·F_2_·2EtOH, the hydrogen atoms from the amino groups of the Hdpa ligand and the amide group of H_2_pia in the complex were assigned based on a difference Fourier map, and the N–H bond lengths were restrained to 0.86(2) Å. The C–C and C–O bond lengths of EtOH with disordered atoms were restrained to 1.52(1) and 1.42(1) Å, respectively. The hydrogen atoms of the ethyl groups of EtOH were located at positions theoretically calculated using the riding model, whereas those of the hydroxy group were assigned based on a difference Fourier map, with the O–H bond lengths restrained to 0.82(2) Å. *U*_iso_(H) was set to 1.5*U*_eq_(O) and 1.2*U*_eq_(C,N) for the hydroxyl and other H atoms, respectively. Highly disordered solvent molecules (presumably volatile diethyl ether) that could not be refined to acceptable levels were also present. Thus, the SQUEEZE routine^34^ in the *PLATON* program^35^ was used to generate a modified dataset in which the contribution of the disordered molecules to the structure amplitudes was discarded. The void volume of 739 Å^3^ occupied by the disordered solvent (20.7% of the unit-cell volume) contained 168 electrons, corresponding to approximately four molecules of the diethyl ether solvent per unit cell. The chemical formulae and other crystal data did not consider unknown solvent molecule(s). The crystallographic data for 1·Cl_2_·2.5H_2_O and 1·F_2_·2EtOH are summarized in Table 1, and the selected bond lengths and angles are listed in Table 2. The crystallographic data have been deposited at the Cambridge Crystallographic Data Center (deposition number CCDC 2167399–2167400).

**Table 1:** Crystallographic data for 1∙Cl_2_·2.5H_2_O and 1·F_2_·2EtOH

	1·Cl_2_·2.5H_2_O	1·F_2_·2EtOH
Chemical formula	RuCl_2_C_26_N_8_O_3.5_H_29_	RuF_2_C_30_N_8_O_3_H_36_
Formula weight	681.54	695.73
Temperature (K)	173	173
Crystal dimensions (mm)	0.30 × 0.10 × 0.05	0.20 × 0.05 × 0.05
Color	Red	Yellow
Crystal system	Triclinic	Monoclinic
Space group	*P-*1 (#2)	*P*2_1_/*c* (#14)
Lattice parameters		
*a* (Å)	9.1020(2)	9.09482(19)
*b* (Å)	10.5348(3)	23.8546(5)
*c* (Å)	17.4020(4)	16.9383(4)
*α* (°)	99.448(2)	90
*β* (°)	102.616(2)	103.492(2)
*γ* (°)	99.741(2)	90
*V* (Å^3^)	1569.40(7)	3573.40(14)
*Z*	2	4
*D*_calcd._ (g cm^−3^)	1.442	1.293
*F*_000_	676.00	1432.00
*μ*(CuKα, cm^−1^)/*μ*(MoKα, cm^−1^)	59.67	4.89
Independent reflections	6216	9765
R1 [*I* > 2σ(*I*)]/No. of reflections	0.0787/5625	0.0322/8060
w*R*_2_ (all data)	0.2118/6216	0.0807/9765
Goodness of fit (GOF)	1.055	1.019

**Table 2:** Selected bond lengths (Å), angles (°), and dihedral angles (°) for 1·Cl_2_·2.5H_2_O and 1·F_2_·2EtOH^a^

	1·Cl_2_·2.5H_2_O	1·F_2_·2EtOH
Ru1–N1	2.078(5)	2.0861(15)
Ru1–N3	2.063(5)	2.0705(15)
Ru1–N4	2.052(5)	2.0517(14)
Ru1–N6	2.079(5)	2.0693(15)
Ru1–N7	2.065(5)	2.0621(15)
Ru1–O1	2.098(4)	2.1058(12)
O1–C26	1.262(8)	1.269(2)
C26–N8	1.326(8)	1.304(2)
C21–C26	1.491(9)	1.496(2)
N1–Ru1–N3	87.40(19)	87.71(6)
N4–Ru1–N6	89.44(19)	90.77(6)
N7–Ru1–O1	78.29(19)	78.16(5)
C1–N2–C6	128.1(5)	127.97(15)
C11–N5–C16	127.9(5)	131.75(16)
O1–C26–N8	121.0(6)	122.09(17)
O1–C26–C21	118.9(5)	117.63(16)
C21–C26–N8	120.1(6)	120.22(17)
Ru1–N1–C1–N2	10.6(8)	−20.1(2)
Ru1–N3–C6–N2	−14.2(7)	5.4(2)
Ru1–N4–C11–N5	−6.4(7)	5.3(2)
Ru1–N6–C16–N5	4.0(8)	−10.7(2)
Plane(1)–Plane(2)	35.0	31.7
Plane(3)–Plane(4)	29.9	15.8

a Plane(1) = N1, C1, C2, C3, C4, C5; Plane(2) = N3, C6, C7, C8, C9, C10; Plane(3) = N4, C11, C12, C13, C14, C15; Plane(4) = N6, C16, C17, C18, C19, C20.

DFT calculations were performed using the Spartan’20 program^36^ on a Macintosh computer. Each structure was fully optimized using the B3LYP functional. Calculations were performed using the LANL2DZ and 6-31G* basis sets for Ru and non-ruthenium atoms, respectively, in vacuum. The stationary points were verified using the results of vibrational analyses.

### 2.2 Synthesis of [Ru(Hdpa)_2_(H_2_pia)]Cl_2_·2.5H_2_O (1·Cl_2_·2.5H_2_O)

A solution of *cis*-[RuCl(dmso-*S*)(Hdpa)_2_]Cl (P1·Cl; 270 mg, 0.45 mmol) and H_2_pia (69 mg, 0.57 mmol) in H_2_O–MeOH (10 mL–10 mL) was refluxed for 3 h, during which the yellow solution turned wine-red within 15 min. The reaction mixture was evaporated to dryness *in vacuo*. The residue was dissolved in MeOH (2 mL), following which diethyl ether (3 mL) and acetone (15 mL) were added. Wine-red crystals of 1·Cl_2_·2.5H_2_O appeared after cooling the solution for a few days, which were collected *via* filtration, washed with acetone, and dried *in vacuo* (280 mg, 91%). Red-brown crystals suitable for X-ray crystallography were obtained by the vapor diffusion of diethyl ether into a methanol solution of 1·Cl_2_·2.5H_2_O. Anal. Calcd for RuCl_2_C_26_N_8_OH_24_·2.5H_2_O: C, 45.81; H, 4.29; N, 16.44%. Found: C, 45.60; H, 4.23; N, 16.62%. ^1^H NMR (500 MHz DMSO-*d*_6_): *δ* = 6.70 (dd, 1H, ^3^*J* = 6.0 and 7.0 Hz, H-5b), 6.78 (m, 2H, H-5a and H-5d), 6.87 (dd, 1H, ^3^*J* = 6.0 and 7.2 Hz, H-5c), 7.36 (d, 1H, ^3^*J* = 8.3 Hz, H-3c), 7.55 (m, 3H, H-3b, H-6a, H-6c), 7.60 (d, 1H, ^3^*J* = 6.0 Hz, H-6b), 7.63 (dd, 1H, ^3^*J* = 7.2 and 8.3 Hz, H-4c), 7.65 (d, 1H, ^3^*J* = 8.8 Hz, H-3a), 7.70 (dd, 1H, ^3^*J* = 7.0 and 8.4 Hz, H-4b), 7.72 (d, 1H, ^3^*J* = 8.8 Hz, H-3d), 7.82 (m, 4H, H-4a, H-4d, H-5e, H-6d), 8.04 (t, 1H, ^3^*J* = 8.0 Hz, H-4e), 8.42 (d, 1H, ^3^*J* = 8.0 Hz, H-3e), 8.91 (d, 1H, ^3^*J* = 4.6 Hz, H-6e), 9.79 (s, 1H, NH), 9.81 (s, 1H, NH), 11.52 (s, 1H, NH), 11.85 (s, 1H, NH).

### 2.3 Synthesis of [Ru(Hdpa)_2_(H_2_pia)](OTf)_2_·H_2_O (1·(OTf)_2_·H_2_O)

An aqueous solution (0.5 mL) of Li(OTf) (185 mg, 1.2 mmol) was added to an aqueous solution (3 mL) of 1·Cl_2_·2.5H_2_O (200 mg, 0.30 mmol), yielding a deep-red precipitate of 1·(OTf)_2_·H_2_O. The precipitate was collected *via* filtration, washed with cold water, and dried *in vacuo* (242 mg, yield 92%). Anal. Calcd for RuF_6_S_2_C_28_N_8_O_7_H_24_·H_2_O; C, 38.14%; H, 2.97%; N, 12.71%. Found: C, 37.95%; H, 2.93%; N, 12.71%. ^1^H NMR (500 MHz DMSO-*d*_6_): *δ* = 6.73 (dd, 1H, ^3^*J* = 6.0 and 7.1 Hz, H-5b), 6.81 (dd, 1H, ^3^*J* = 6.0 and 7.2 Hz, H-5a), 6.82 (dd, 1H, ^3^*J* = 5.9 and 7.2 Hz, H-5d), 6.89 (dd, 1H, ^3^*J* = 6.0 and 7.1 Hz, H-5c), 7.00 (d, 1H, ^3^*J* = 8.3 Hz, H-3c), 7.17 (d, 1H, ^3^*J* = 8.2 Hz, H-3b), 7.30 (d, 2H, ^3^*J* = 8.4 Hz, H-3a and H-3d), 7.54 (d, 1H, ^3^*J* = 6.0 Hz, H-6c), 7.60 (d, 1H, ^3^*J* = 6.0 Hz, H-6a), 7.62 (d, 1H, ^3^*J* = 6.0 Hz, H-6b), 7.65 (dd, 1H, ^3^*J* = 7.1 and 8.3 Hz, H-4c), 7.73 (dd, 1H, ^3^*J* = 7.1 and 8.2 Hz, H-4b), 7.81 (m, 2H, H-5e and H-6d), 7.85 (dd, 1H, ^3^*J* = 7.2 and 8.4 Hz, H-4d), 7.86 (dd, 1H, ^3^*J* = 7.2 and 8.4 Hz, H-4a), 8.06 (dd, 1H, ^3^*J* = 7.7 and 7.9 Hz, H-4e), 8.19 (d, 1H, ^3^*J* = 7.9 Hz, H-3e), 8.94 (d, 1H, ^3^*J* = 5.4 Hz, H-6e), 9.50 (s, 1H, NH), 9.68 (s, 1H, NH), 10.62 (s, 1H, NH), 10.74 (s, 1H, NH). ^1^H NMR (500 MHz CD_3_CN): *δ* = 6.66 (dd, 1H, ^3^*J* = 6.0 and 7.1 Hz, H-5b), 6.72 (m, 2H, H-5a and H-5d), 6.80 (dd, 1H, ^3^*J* = 6.0 and 7.1 Hz, H-5c), 7.01 (d, 1H, ^3^*J* = 8.3 Hz, H-3b), 7.16 (d, 1H, ^3^*J* = 8.3 Hz, H-3c), 7.30 (d, 1H, ^3^*J* = 8.4 Hz, H-3a), 7.32 (d, 1H, ^3^*J* = 8.0 Hz, H-3d), 7.54 (d, 1H, ^3^*J* = 6.0 Hz, H-6b), 7.59 (m, 3H, H-4b, H-6c, NH), 7.67 (m, 3H, H-4c, H-5e, H-6a), 7.78 (m, 3H, H-4a, H-4d, H-6c), 7.90 (d, 1H, ^3^*J* = 7.8 Hz, H-3e), 7.94 (dd, 1H, ^3^*J* = 6.9 and 7.9 Hz, H-4e), 7.99 (s, br, 1H, NH), 8.92 (d, 1H, ^3^*J* = 5.4 Hz, H-6e), 9.05 (s, 1H, NH), 9.23 (s, 1H, NH).

### 2.4 Synthesis of [Ru(Hdpa)_2_(H_2_pia)]F_2_·4H_2_O (1·F_2_·4H_2_O) using 1·(OTf)_2_·H_2_O and tetra-*n*-butylammonium fluoride (TBAF)

An acetonitrile solution (2.5 mL) of tetra-*n*-butylammonium fluoride trihydrate (TBAF·3H_2_O; 298 mg, 0.94 mmol) was added to an acetonitrile solution (2.5 mL) of 1·(OTf)_2_·H_2_O (330 mg, 0.38 mmol), yielding a dark-brown precipitate of 1·F_2_·4H_2_O. The precipitate obtained after cooling the reaction mixture was washed three times *via* decantation with a small amount of cold acetonitrile, and diethyl ether (10 mL) was added to the residue. The precipitate was collected *via* filtration, washed with diethyl ether, and dried *in vacuo* (220 mg, 87%). Anal. Calcd for RuF_2_C_26_N_8_OH_24_·4H_2_O; C, 46.21%; H, 4.77%; N, 16.59%; F, 5.62. Found: C, 46.48%; H, 4.87%; N, 16.65%; F, 5.48%. Yellow crystals of 1·F_2_·2EtOH, suitable for X-ray crystallography, were obtained by the vapor diffusion of diethyl ether into an ethanol solution of 1·F_2_·4H_2_O.

## 3 Results and discussion

### 3.1 Synthesis of 1·Cl_2_ and anion exchange with OTf^−^ or F^−^

The reaction between *cis*-[RuCl(dmso-*S*)(Hdpa)_2_]Cl (P1·Cl) and H_2_pia *via* reflux in a mixed solution (water–MeOH = 1 : 1 v/v) afforded the deep red complex [Ru(Hdpa)_2_(H_2_pia)]Cl_2_·2.5H_2_O (1·Cl_2_·2.5H_2_O) in high yield (91%). Triflate salt 1·(OTf)_2_·H_2_O, which was also a deep red precipitate, was quantitatively obtained by adding a suitable amount of Li(OTf) to an aqueous solution of 1·Cl_2_·2.5H_2_O. The chloride anions interact *via* hydrogen bonding with amino or amide protons of complexes in solution and crystals. Whereas, the triflate anions interact *via* very weak interactions with amino or amide protons in solution and in crystals. In general, chloride salt can be used to obtain suitable crystals for X-ray analysis instead of using the triflate salt. Moreover, with regard to the absorption and NMR spectroscopic data analyses, the interaction between triflate anions and 1^2+^ is excessively weak, such that it does not interfere with the interaction between 1^2+^ and fluoride anions. Furthermore, although chloride anions interact with 1^2+^, this interaction is weak and does not interfere with the interaction with fluoride.

The subsequent addition of TBAF to an acetonitrile solution of 1·(OTf)_2_·H_2_O caused the red solution to turn yellow and yield a dark brown precipitate. The change in the color of the solution suggested that the reaction of 1·(OTf)_2_·H_2_O with F^−^ anions afforded a deprotonated dpa^−^ species with HF or a fluoride adduct species F–Hdpa. The ^1^H NMR spectrum of 1·F_2_·4H_2_O in DMSO-*d*_6_ (Fig. S5†) showed a total of 20 H signals in the *δ* region of 6.3–9.0, suggesting that the dark brown precipitate had five pyridyl groups and the sample was of high purity. However, neither the amino NH nor the amide NH_2_ signals were observed in the spectrum, and an extremely broad signal representing H_2_O appeared. Therefore, the dark brown precipitate could be either a deprotonated dpa^−^ complex [Ru(dpa^−^)_2_(H_2_pia)] or fluoride salt [Ru(Hdpa)_2_(H_2_pia)]F_2_. Elemental CHNF analysis of the dark brown precipitate indicated the occurrence of an anion-exchange reaction and confirmed the dark-brown product to be a fluoride salt, that is, [Ru(Hdpa)_2_(H_2_pia)]F_2_·4H_2_O (1·F_2_·4H_2_O). Single crystals of the fluoride salt, 1·F_2_·2EtOH, were obtained by vapor diffusion of diethyl ether into an ethanol solution of 1·F_2_·4H_2_O. The single-crystal structure of the fluoride salt also confirmed its presence as a [Ru(Hdpa)_2_(H_2_pia)]^2+^ dication with two F^−^ anions, as explained later in Section 3.2.

In our previous research, the corresponding bis-bpy fluoride-salt complex, [Ru(bpy)_2_(H_2_pia)]F_2_·6H_2_O (P2·F_2_·6H_2_O) was soluble in acetonitrile; therefore, to ensure its isolation, diethyl ether was added to an acetonitrile solution of P2·(OTf)_2_ with TBAF.^30^ However, the fluoride salt 1·F_2_·4H_2_O reported herein was poorly soluble in acetonitrile. Essentially, 1·F_2_·4H_2_O spontaneously precipitated from the acetonitrile solution without diethyl ether. Moreover, the fluoride salt 1·F_2_·4H_2_O was soluble in water, methanol, and ethanol. To recover the 1^2+^ and F^−^ ions of 1·F_2_·4H_2_O, equivalents of Li(OTf) were added to an aqueous solution of 1·F_2_·4H_2_O, and the deep red 1·(OTf)_2_·H_2_O solid was precipitated from the solution. The recovery of 1^2+^ was ∼90%, and the fluoride ions remained in the solution as LiF, which suggested that the fluoride-sensing complex 1·(OTf)_2_·H_2_O could be reused. This could be important for achieving sustainable development goals.

### 3.2 Crystal structures of synthesized chloride and fluoride salts

**Fig. 1:**
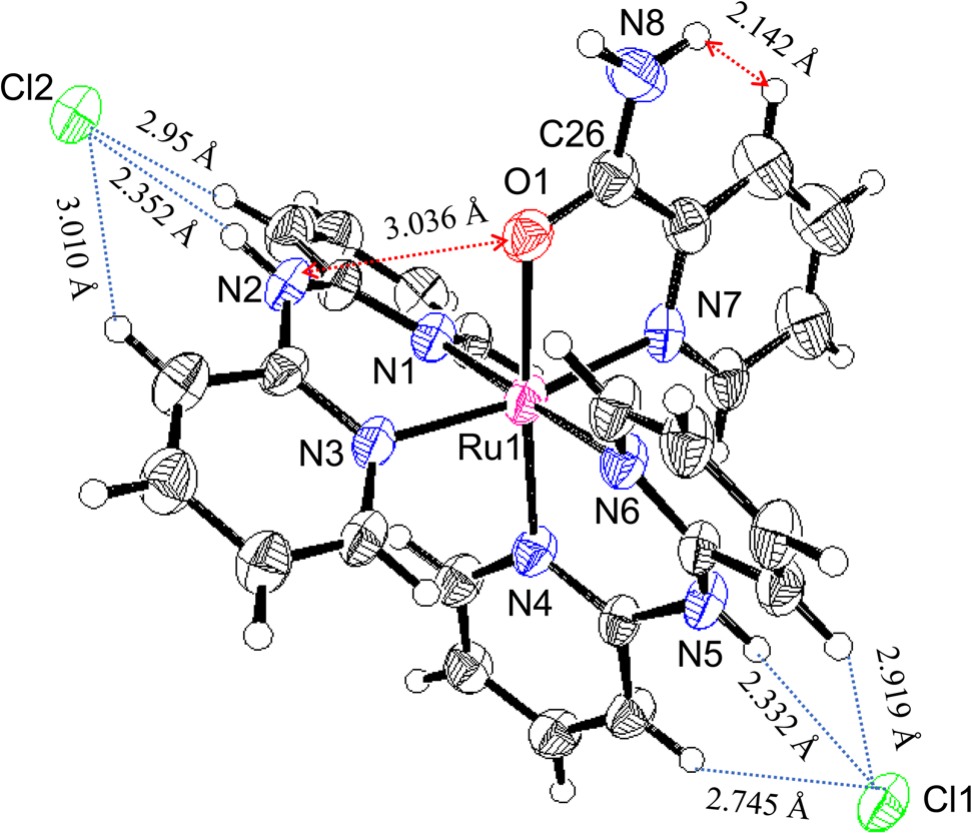
ORTER drawing of 1·Cl_2_·2.5H_2_O Ellipsoids are drawn at 50% probability level.

Oak Ridge thermal ellipsoid plot (ORTEP) drawings of 1·Cl_2_·2.5H_2_O and 1·F_2_·2EtOH were constructed (Fig. 1 and S6,† respectively). The complex cations in the chloride and fluoride salts were identical, with no rearrangement of the coordination sphere around Ru ions. Both Ru ions had a slightly distorted octahedral geometry with two Hdpa ligands and an H_2_pia ligand; two Hdpa coordinated with the Ru^2+^ ion *via* two pyridyl-N atoms, and H_2_pia coordinated with the Ru^2+^ ion *via* pyridyl-N and amide-O atom.

**Fig. 2:**
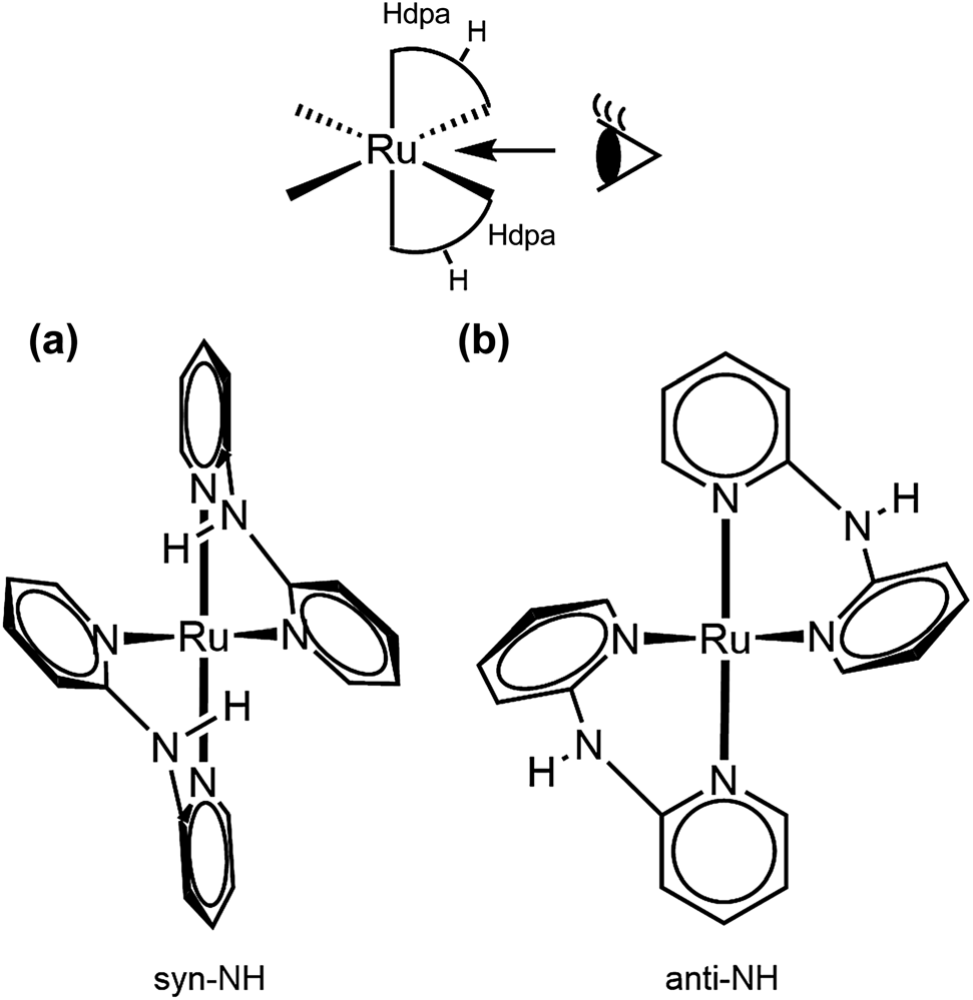
Two types of Hdpa conformations in bis(Hdpa)Ru(ii) complexes: (a) *syn*-NH and (b) *anti*-NH.

The two Hdpa ligands in both complexes had bent conformations, although the degree of bending of the Hdpa ligands in both complexes was different; these details are provided later. Two pyridyl groups in the bent Hdpa(*N*1,*N*3) ligand captured the pyridyl-N4 group of another bent Hdpa(*N*4,*N*6) ligand, whose two pyridyl groups captured the pyridyl-N3 group. The combination of two bent conformations of the Hdpa ligands compelled both NH groups in each Hdpa ligand to point in opposite directions (*anti*-NH conformation, Fig. 2b). The combination of conformations of the two Hdpa ligands in 1^2+^ has also been observed in [Ru(bpy)(Hdpa)_2_](ClO_4_)_2_,^18^ but not in the starting complex *cis*-[RuCl(dmso-*S*)(Hdpa)_2_]^+^ (P1^+^).^29^ Each Hdpa ligand in P1^+^ captured a monodentate ligand, Cl, or a methyl group of the dmso-*S* ligands using two pyridyl groups, resulting in both NH groups being close to each other (*syn*-NH conformation, Fig. 2a). Bulky dmso-*S* and Cl^−^ ligands lead to the *syn*-NH conformation of P1^+^, whereas planar slime H_2_pia and planar bpy ligands result in its anti-NH conformation. This combination of conformations in bis(Hdpa)Ru(ii) complexes could affect the reaction between the NH groups and F^−^ anions in solution.

The lengths of four Ru–N bonds between the Ru1 and four pyridyl-N (N1, N3, N4, and N6) of two Hdpa ligands in 1·Cl_2_·2.5H_2_O were similar to those in 1·F_2_·2EtOH, suggesting that these Ru–N bond lengths between the Ru1 and four pyridyl-N of two Hdpa ligands in 1^2+^ were less sensitive to the counter-ions and solvent molecules (Table 2). Among these, the Ru1–N4 bond length [2.052(5) and 2.0517(12) Å in 1·Cl_2_·2.5H_2_O and 1·F_2_·2EtOH, respectively] was the lowest, probably because of the coordination of the O atom of H_2_pia *trans* to N4. The Ru1–N7(H_2_pia) bond lengths [2.065(5) and 2.0621(15) Å in 1·Cl_2_·2.5H_2_O and 1·F_2_·2EtOH, respectively] were similar to the corresponding bond lengths in [Ru(bpy)_2_(H_2_pia)]Cl_2_ [P2·Cl_2_; 2.057(2) Å]. Moreover, the Ru1–O1(H_2_pia) bond lengths [2.098(4) and 2.1058(12) Å in 1·Cl_2_·2.5H_2_O and 1·F_2_·2EtOH, respectively] were also similar to the corresponding Ru–O(H_2_pia) bond length in P2·Cl_2_ [2.090(2) Å].^30^

The structural parameters around the C26 atom in the amide group of the H_2_pia ligand in the chloride salt were similar to those in the fluoride salt and P2·Cl_2_; however, they were slightly different from those in free H_2_pia.^30,37^ The O1–C26 bond lengths in the Ru(ii)–H_2_pia complexes [1.262(8), 1.269(2), and 1.260(3) Å in 1·Cl_2_·2.5H_2_O, 1·F_2_·2EtOH, and P2·Cl_2_, respectively] were greater than those in free H_2_pia (1.24 Å). Moreover, the O1–C26 bond lengths in the Ru(ii)–H_2_pia complexes were between those of C–O^−^ and C=O in [Ru(bpy)_2_(pic)]Cl (pic = picolinato) [1.222(3) and 1.294(3) Å, respectively].^38^ Thus, the O1–C26 bond lengths in Ru(ii)–H_2_pia complexes, P2^2+^ and 1^2+^, were closer to C–O^−^ in comparison with the C=O bond length. So that, the coordinated O1 in these complexes was slightly negatively charged. The C21–C26 bond lengths in the Ru(ii)–H_2_pia complexes [1.491(9), 1.496(2), and 1.494(4) Å in 1∙Cl_2_·2.5H_2_O, 1·F_2_·2EtOH, and P2·Cl_2_, respectively] were slightly lower than those in free H_2_pia and [Ru(bpy)_2_(pic)]Cl [1.51 and 1.508(3) Å, respectively]. Moreover, the C26–N8 bond lengths in the Ru(ii)–H_2_pia complexes [1.326(8), 1.304(2), and 1.312(4) Å in 1·Cl_2_·2.5H_2_O, 1·F_2_·2EtOH, and P2·Cl_2_, respectively] were slightly lower than those in free H_2_pia (1.33 Å). These results indicate that the π-electron of the carbonyl group of H_2_pia in the Ru(ii)–H_2_pia complexes was delocalized over the entire amide group.

The major structural difference between the synthesized chloride and fluoride salts is in the conformation of Hdpa. The bite angles of the Hdpa ligands [87.40(19)° and 89.44(19)° in the chloride salt; 87.71(6)° and 90.77(6)° in the fluoride salt] were nearly identical and close to the ideal angle in octahedral complexes (90°). However, the dihedral angles in both complexes, which are the angles between the two pyridyl groups in the Hdpa ligand, were related to ligand/ligand or NH/anion interactions. For the chloride salt, the dihedral angle for Hdpa(*N*1,*N*3) was greater than that for Hdpa(*N*4,*N*6) (35.0° and 29.9°, respectively). Both angles were consistent with those of the Hdpa ligand in previously reported Ru(ii)–Hdpa complexes (25.9–48.2°).^18,29,39,40^ The difference between the two dihedral angles is likely caused by steric interactions between H_2_pia and the two Hdpa ligands. The N2 atom of the NH group in Hdpa(*N*1,*N*3) established contact with the O1 atom of H_2_pia (N2⋯O1 = 3.036 Å; Fig. 1 and S7a†). In contrast, the closest distance between Hdpa(*N*4,*N*6) and H_2_pia was achieved *via* contact between C11 in Hdpa(*N*4,*N*6) and H-6 (H25) of the pyridyl group in H_2_pia (C11⋯H25 = 2.757 Å; Fig. 1 and S7c†). Therefore, Hdpa(*N*1,*N*3) established contact with the slime O1 site of H_2_pia and exhibited a more bent conformation (35.0°). Hdpa(*N*4,*N*6) linked to the bulky pyridyl site of H_2_pia and had a less bent conformation (29.9°). H_2_pia, which is unsymmetrical and forms a rigid five-membered chelate ring with a Ru^2+^ ion, affects the dihedral angles of the Hdpa ligands in the complex. The Hdpa ligand can accommodate the steric demand from other ligands in a complex to alter its conformation.

In the fluoride salt, the dihedral angle for Hdpa(*N*1,*N*3) was slightly smaller than that in the chloride salt (31.7° and 35.0°, respectively). Moreover, the dihedral angle for the other Hdpa(*N*4,*N*6) was significantly smaller than that in the chloride salt (15.8° and 29.9°, respectively), and smaller than previously reported Ru(ii)–Hdpa data (25.9°–48.2°).^18,29,39,40^ Generally, the bent conformation of Hdpa cuts off a π-conjugated system between two pyridyl groups in the Hdpa ligand. However, the smaller dihedral angle of Hdpa(*N*4,*N*6) indicated that this Hdpa possessed the π-conjugated system of the entire Hdpa ligand, similar to a coordinated bpy ligand. The color of complex [Ru(bpy)_3_]^2+^, in which the bpy ligand is formed as a flat five-membered chelate ring, is red. However, the color of [Ru(Hdpa)_3_]^2+^, in which the Hdpa ligand is formed as a bent six-membered chelate ring, is yellow.^18^ Therefore, the intense dark brown color of the fluoride salt suggests that the π-conjugated system of the entire Hdpa ligand was extended and stabilized.

In the crystal of the chloride salt, each NH group of both Hdpa ligands formed hydrogen bonds with the two Cl^−^ anions (Fig. 1 and Table S1†), and the H-3 protons in the pyridyl group in both Hdpa ligands also established links with the Cl^−^ anions (Fig. S7b†). The N5–H5A atoms of the Hdpa(*N*4,*N*6) ligand exhibited hydrogen-bonding interactions with the counterion Cl1 (H5A⋯Cl1; 2.332 Å) and two weak hydrogen-bonding interactions with the pyridyl H-3 protons (H12⋯Cl1 and H17⋯Cl1; 2.745 and 2.919 Å, respectively). The N2–H2A atoms of the Hdpa(*N*1,*N*3) ligand formed a hydrogen bond with another counterion, Cl2 (H2A⋯Cl2; 2.352 Å), and formed two weak hydrogen bonds between Cl2 and two pyridyl H-3 protons (H2⋯Cl2 and H7⋯Cl2; 2.954 and 3.010 Å, respectively). Moreover, the counterion Cl2 was connected to the proton of the amide group, H8B, and the H-3 proton of the pyridyl–N7 linkage in the H_2_pia ligand of the neighboring complex (H8B⋯Cl2 and H22⋯Cl2; 2.542 and 2.869 Å, respectively). Therefore, two 1^2+^ ions were connected by two Cl2 atoms to form a dimer structure (Fig. S8a†). These dimers were observed along the *a-*axis (Fig. S8b†).

The packing and location of the two counter-anions in the fluoride salt were different from those in the chloride salt. In the crystal of the fluoride salt, the N2–H2A atoms of the Hdpa(*N*1,*N*3) ligand exhibited hydrogen-bonding interactions with the counterion F2 (H2A⋯F2; 1.740 Å) and two weak hydrogen-bonding interactions with pyridyl H-3 (H2⋯F2 and H7⋯F2; 2.653 and 2.441 Å, respectively). Moreover, the F2 atom exhibited hydrogen-bonding interactions with the N5–H5A atoms of the Hdpa(*N*4,*N*6) ligand in the neighboring complex (H5A⋯F2; 1.740 Å) and two weak hydrogen-bonding interactions with pyridyl H-3 (H12⋯F2 and H17⋯F2; 2.487 and 2.517 Å, respectively) (Fig. S9a†). Therefore, 1^2+^ connected to F2 atoms and formed a 1D hydrogen-bonding chain along the *c*-axis (Fig. S10†). The other counterion, F1, was connected to the proton of the amide group, H8B, and the H-3 proton of pyridyl-N7 (H8B⋯F1 and H22⋯F1; 1.770 and 2.335 Å, respectively) (Fig. S9b†). The two EtOH molecules in the crystal formed hydrogen bonds with the F1 ion *via* the proton of the hydroxy group (H2E⋯F1 and H3E⋯F1; 1.722 and 1.724 Å, respectively). F1 had a slightly distorted tetrahedral geometry with three strong and one weak hydrogen bonds. The O2 in the ethanol molecule formed a hydrogen bond with the remaining amide proton, H8B, in another 1D chain. Thus, the two EtOH molecules and two amides between two 1D chains formed a hexagonal small hole *via* hydrogen bonding (Fig. S11†). Consequently, in the fluoride salt crystal, the 1D hydrogen-bonding chains with F2 atoms underwent crosslinking with F1-containing hexagonal hydrogen bonding, forming a 3D network. The complex dication 1^2+^ was surrounded by three F^−^ ions that interacted with two NH groups and an amide NH group (Fig. S6†). This crystal structure corresponds exactly to that of tris-F-adduct-1^2+^, 1·F_3_^−^, in solution, which is discussed further in Section 3.5. Voids with a volume of 370 Å^3^ and centered at (0.002, 0.500, 0.000) and (0.004, 0.000, 0.500), each containing 85 electrons, were present in these 3D networks (Fig. S12†). An additional electron density of 170 electrons per unit cell corresponds to approximately four molecules of diethyl ether solvent (42 electrons). However, diethyl ether could not be refined to an acceptable level. Thus, the SQUEEZE routine^34^ in the *PLATON* program^35^ was used, and an Et_2_O-solvent-free model was employed for the final refinement.

### 3.3 ^1^H NMR analysis of 1·X_2_ (X = Cl or OTf)

**Fig. 3:**
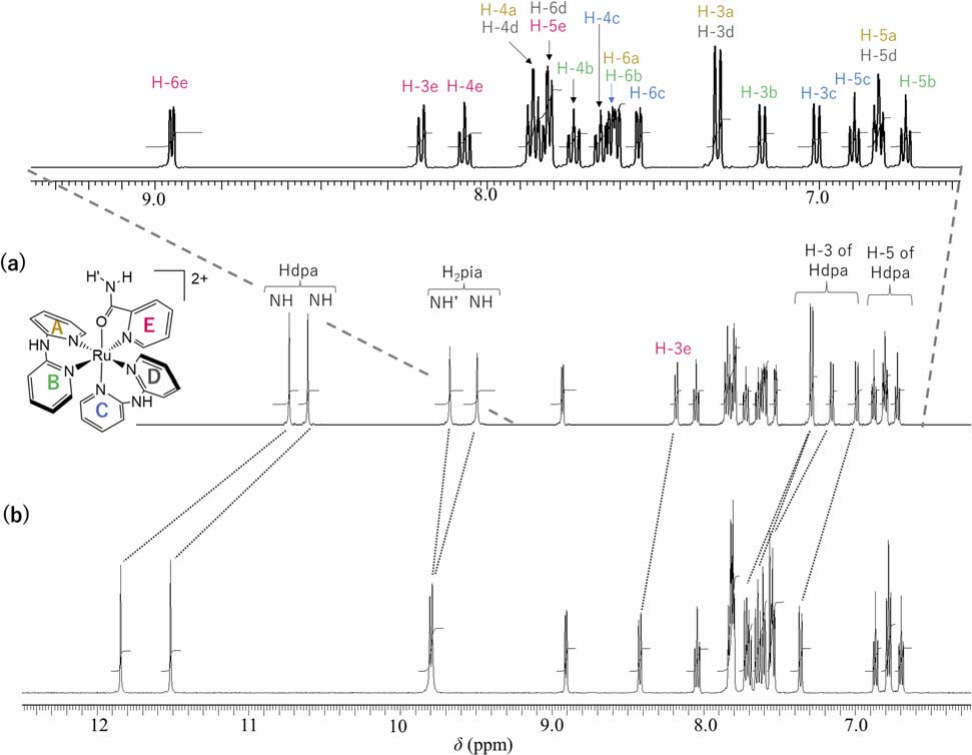
^1^H NMR spectra (500 MHz, 298 K) of (a) 1·(OTf)_2_·H_2_O and (b) 1·Cl_2_·2.5H_2_O in DMSO-*d*_6_.

The ^1^H NMR spectra of 1·(OTf)_2_·H_2_O and 1·Cl_2_·2.5H_2_O in DMSO-*d*_6_ are shown in Fig. 3. In the spectrum of 1·(OTf)_2_·H_2_O (Fig. 3a), four singlets corresponding to the NH groups appear at *δ* 10.74, 10.62, 9.68, and 9.50. Based on their chemical shifts, two of them at a lower field (*δ* 10.74 and 10.62) were assigned to two NH groups of each of the coordinated Hdpa ligands in 1^2+^, because the NH signals of Hdpa ligands in *cis*-[RuCl(dmso-*S*)(Hdpa)_2_](OTf) (P1·(OTf)) in DMSO-*d*_6_ were observed at *δ* 10.32 as a singlet signal with 2H intensities at a lower field than that of two NH signals of the amide protons of the H_2_pia ligand in [Ru(bpy)_2_(H_2_pia)](OTf)_2_ (P2·(OTf)_2_) in DMSO-*d*_6_ (*δ* 9.98 and 9.82).^29,30^ Therefore, the remaining two singlets in the high field (*δ* 9.68 and 9.50) were assigned to two protons in the amide group of H_2_pia, indicating that the two protons of the amide group were in different environments, similar to the amide protons in the ^1^H NMR spectrum of P2·(OTf)_2_.^30^ This indicated that the rotation around the C–N bond of the amide group of H_2_pia in 1^2+^ was fixed in the solution. Thus, the structural feature of the amide group in the H_2_pia ligand in 1^2+^ that was observed in the crystal structure was maintained even in the solution. In the *δ* region of 6.7–9.0, a total of 20 H signals were observed, which corresponded to the number of protons in the five pyridyl groups. The 20 signals of the pyridyl groups were classified into five sets of pyridyl protons based on their coupling constants and ^1^H–^1^H COSY analysis.^41^ The five sets were assigned to the A–E pyridyl rings based on the distinctive signals of each set (Fig. 3; Tables S2 and S3†).

The H-3 signal at *δ* 8.19 was observed at a significantly lower field compared to the other four H-3 signals (*δ* 7.00–7.30), which was characteristic of the proton at the 3-position of the pyridyl group in H_2_pia; thus, the signal was assigned to H-3 of the E-ring in 1^2+^ (H-3e). The pyridyl group of the C-ring, in which the Ru1–N4 bond is shorter than other Ru–N(Hdpa) bonds (see crystal structure), can accept an electron from a Ru^2+^ ion *via* π-back-donation, enabling detection of the protons of the C-ring at a relatively higher field. In particular, this tendency could be found in the H-4 signal, which corresponded to the *para* position to the donor N atom in the pyridine ring. The H-4 signal at *δ* 7.65 appeared at the highest field compared to the other H-4 signals (*δ* 7.73–7.86), following which the signal was assigned to the C-ring (H-4c). The H-6 signal at *δ* 7.81 was observed at a lower field compared to the other three H-6 signals (*δ* 7.54–7.62). Although the H-6 protons of the A-, B-, and C-rings were in the vicinity of other pyridyl groups and suffered a high field shift owing to the ring-current effect, the H-6 proton of the D-ring migrated to the amido group of the H_2_pia ligand and suffered no ring-current effect. Thus, the H-6 signal at *δ* 7.81 was assigned to the H-6 proton in the D-ring (H-6d). In the ^1^H NMR spectrum of P2·(OTf)_2_, the corresponding H-6 signal was also observed at a lower field compared to the other H-6 signals.^30^ The protons in the A-ring were in environments similar to those in the D-ring, except for the ring-current effect, because both were *trans* to the pyridyl groups of the Hdpa ligands. Thus, the protons of the set (*δ* 6.81, 7.30, 7.60, and 7.86), which showed similar chemical shifts to those of the D-ring except for the H-6 signals, were in the A-ring (Fig. S13 and S14†), and the remaining protons of the set were in the B-ring.

The ^1^H NMR spectrum of the chloride salt 1·Cl_2_·2.5H_2_O in DMSO-*d*_6_ (Fig. 3b) was slightly different from that of the triflate salt 1·(OTf)_2_·H_2_O (Fig. 3a). For 1·Cl_2_·2.5H_2_O, the two NH signals corresponding to the Hdpa ligands in the complex (*δ* 11.52 and 11.85) were significantly shifted to a lower field compared to the signals in 1·(OTf)_2_·H_2_O (*δ* 10.62 and 10.74). This suggested that the Cl^−^ counterion interacted with the NH groups of the Hdpa ligands in 1^2+^ to form N–H⋯Cl^−^ hydrogen-bonding interactions, which were observed in the crystal structure of 1·Cl_2_·2.5H_2_O (Fig. 1). Moreover, the two signals of amide protons (*δ* 9.79 and 9.81) and that of the H-3e proton (*δ* 8.42) of 1·Cl_2_·2.5H_2_O shifted to lower fields than those of the counterparts in 1·(OTf)_2_·H_2_O (*δ* 9.50, 9.68, and 8.19, respectively). This indicated that the Cl^−^ counter-ions also established contacts with the amide proton and H-3 proton of H_2_pia, and N–H⋯Cl^−^···H-3 hydrogen-bonding interactions were formed, as in the chloride salt crystal (Fig. S8†).^30^

**Fig. 4:**
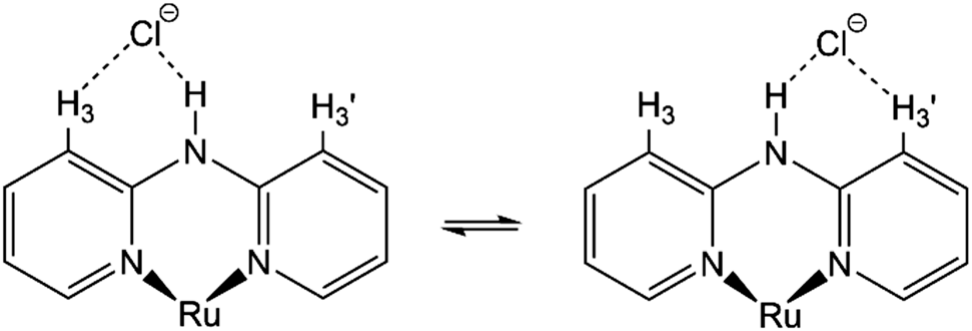
Proposed structures showing hydrogen-bonding interactions between a Cl^−^ anion and the Ru(ii)–Hdpa complex.

Moreover, although other signals (H-4, H-5, and H-6) of the Hdpa ligand in 1·Cl_2_·2.5H_2_O were observed at the same chemical shift as in 1·(OTf)_2_·H_2_O (Fig. 3 and Table S3†), the remaining four H-3 signals also slightly shifted to a lower field. The shift for only the H-3 signals of the Hdpa ligand, due to Cl^−^ anions, was also observed in the ^1^H NMR spectra of P1·X (X = Cl and OTf) in DMSO-*d*_6_.^29^ The N–H⋯Cl^−^ hydrogen-bonding interactions also influenced the H-3 signals, suggesting that the N–H⋯Cl^−^···H-3 or N–H⋯Cl^−^⋯H-3′ hydrogen-bonding interactions alternated (Fig. 4). Although the interactions of the Cl^−^ anion with NH and H-3 protons were not considerably strong, all interactions occurred to the same degree, indicating the flexibility of the hydrogen bonds.

Although the four NH singlets were observed as sharp singlets in DMSO-*d*_6_, the ^1^H NMR spectrum of 1·(OTf)_2_·H_2_O in CD_3_CN showed two sharp singlets and one broad singlet at *δ* 9.23, 9.05, and 7.99, respectively (Fig. S13c†); the remaining NH signal was unclear because certain pyridyl signals overlapped at *δ* ∼ 7.6. The two broad NH signals at a higher field corresponded to the amide protons in H_2_pia, and the two intense NH signals at a lower field corresponded to the NH protons in each Hdpa ligand in 1·(OTf)_2_·H_2_O. The chemical shifts of the NH signals are consistent with those in P2·(OTf)_2_, P1·(OTf), and [Ru(bpy)(Hdpa)_2_](ClO_4_)_2_ in CD_3_CN.^22,29,30^ All four NH signals were shifted to a higher field compared to those in DMSO-*d*_6_. This indicated that all amino and amide protons interacted with the solvent DMSO molecules.

### 3.4 Recognition of fluoride in DMSO by absorption spectroscopy

**Fig. 5:**
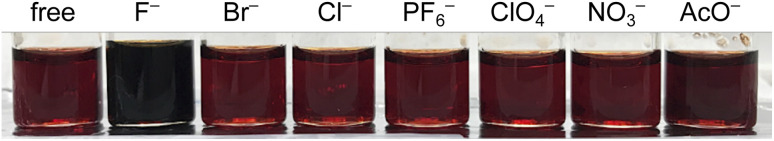
Color changes observed when various TBA salts with anions were added to a DMSO solution of 1·(OTf)_2_·H_2_O. The “free” solution on the far left corresponds to only 1·(OTf)_2_·H_2_O dissolved in DMSO.

A photograph of 1·(OTf)_2_·H_2_O in DMSO with tetra-*n*-butylammonium (TBA) salts—F^−^, Br^−^, Cl^−^, PF_6_^−^, ClO_4_^−^, NO_3_^−^, and CH_3_COO^−^ (AcO^−^)—is shown in Fig. 5. Upon the addition of TBAF, the red DMSO solution of 1·(OTf)_2_·H_2_O turned dark brown, which was visible to the naked eye. Similarly, the addition of TBAF to the DMSO solution of the chloride salt 1·Cl_2_·2.5H_2_O turned the red solution dark brown. This indicated that the chloride anion did not obstruct the interactions between 1^2+^ and the fluoride anion, although the ^1^H NMR analysis of 1·Cl_2_·2.5H_2_O showed that Cl^−^ anions exhibited hydrogen-bonding interactions with an amide and two amino protons in solution. It was highlighting the feasibility of 1·Cl_2_·2.5H_2_O as a fluoride sensor, similar to 1·(OTf)_2_·H_2_O.

**Fig. 6:**
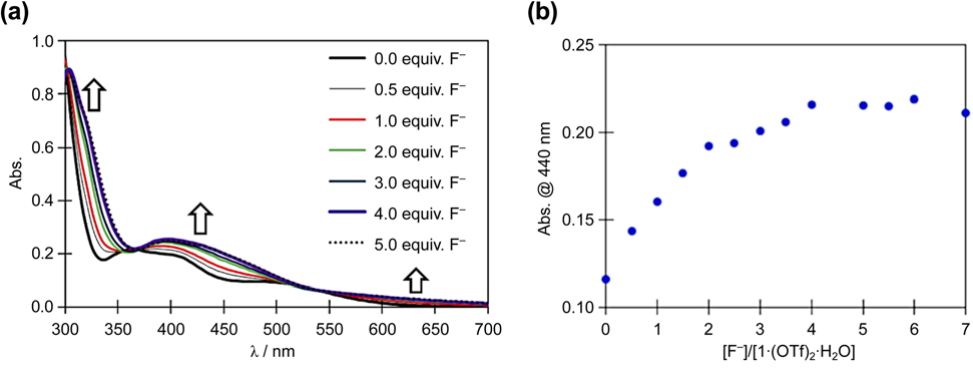
(a) Family of absorption spectra collected during titration of a 2.0 × 10^−5^ M DMSO solution of 1·(OTf)_2_·H_2_O with a standard solution of TBAF. (b) Titration profiles at 440 nm.

The reaction of 1·(OTf)_2_·H_2_O with the F^−^ anion in DMSO was monitored by UV-vis absorption spectroscopy (Fig. 6). After titrating a DMSO solution of TBAF into a DMSO solution of 1·(OTf)_2_·H_2_O, bands appeared at approximately 450 and 320 nm, along with less intense bands at 625 nm. Surprisingly, isosbestic points were observed at ∼365 and 525 nm in the absorbance spectra upon the addition of F^−^ anions, because 1^2+^ had two NH in each of the Hdpa ligands and two protons in the amide group in the H_2_pia ligand; the amino and amide protons might be accepted owing to the addition of F^−^ anions. In our previous study on *cis*-[RuCl(dmso-*S*)(Hdpa)_2_](OTf) (P1·(OTf)) and [Ru(bpy)_2_(H_2_pia)](OTf)_2_ (P2·(OTf)_2_), isosbestic points were not observed in the corresponding absorption spectra, and their spectra indicated the occurrence of two-step reactions with F^−^ anions.^20,30^ The spectrophotometric titration results reported herein are consistent with those of the ^1^H NMR spectra. The next section on NMR analysis of fluoride recognition in DMSO provides more details regarding the reaction between 1^2+^ and F^−^ anions.

The absorption spectrum of 1·Cl_2_·2.5H_2_O in DMSO was almost identical to that of 1·(OTf)_2_·H_2_O in DMSO (Fig. S15†), and the interactions of Cl^−^ anions with 1^2+^ were not detected in the absorption spectra, although they were detected in the NMR spectra of 1·Cl_2_·2.5H_2_O in DMSO. Moreover, titration of 1·Cl_2_·2.5H_2_O with F^−^ in DMSO (Fig. S16†) yielded similar results to those of 1·(OTf)_2_·H_2_O (Fig. 6), indicating that the presence of the Cl^−^ counter-anion did not obstruct the reactions of 1·Cl_2_·2.5H_2_O with F^−^ anions.

Based on the spectrophotometric titration for quantitative analysis of F^−^, the detection limits for F^−^ were calculated to be ∼0.68 × 10^−5^ and ∼0.89 × 10^−5^ M using 1·(OTf)_2_·H_2_O (Fig. S17†) and 1·Cl_2_·2.5H_2_O (Fig. S18†), respectively.^42,43^ Upon the addition of a suitable amount of Li(OTf) to the dark brown TBAF-containing solution of 1·(OTf)_2_·H_2_O or 1·Cl_2_·2.5H_2_O, the solution transformed into the original red solution, whose absorption spectrum was identical to that of 1·(OTf)_2_·H_2_O. The Li^+^ cation bonded to the F^−^ anion to form LiF and regenerated 1^2+^. This masking effect of the Li^+^ cation on the F^−^ anion was observed in our previous research on Ru(ii)–Hdpa and the Ru(ii)–H_2_pia complexes.^29,30^

### 3.5 NMR investigation of reaction between 1·X_2_ (X = OTf or Cl) and TBAF in DMSO-*d*_6_

**Fig. 7:**
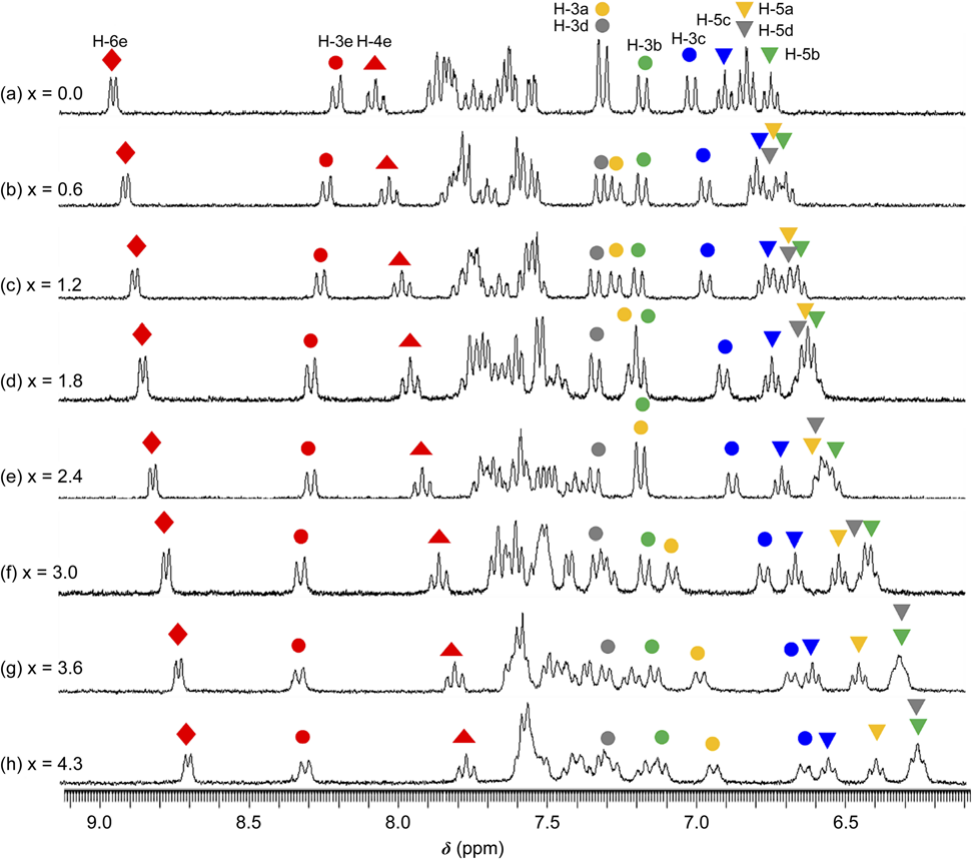
^1^H NMR spectra collected during the reaction between a DMSO-*d*_6_ solution of 1·(OTf)_2_·H_2_O (3.2 × 10^−2^ M) and TBAF. *x* = [TBAF]/[1·(OTf)_2_·H_2_O] (300 MHz NMR; 298 K).

The ^1^H NMR spectra of 1·(OTf)_2_·H_2_O in DMSO-*d*_6_ undergoing titration with TBAF were acquired (Fig. 7). Upon the addition of a small amount of TBAF (*x* = [TBAF]/[1^2+^] = 0.6), four NH signals were not observed because of their transformation into considerably broad signals, possibly suggesting that the amide and two NH groups interacted with the F^−^ anion owing to a rapid exchange and large difference in chemical shift between the F^−^ adduct and free complex cation. The loss of NH signals has also been observed in the ^1^H NMR spectrum of *cis*-[RuCl(dmso-*S*)(Hdpa)_2_](OTf) (P1·(OTf)) that included F^−^ anions in DMSO-*d*_6_.^29^ As the amount of TBAF added increased, the H-4e (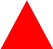) and H-6e (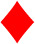) signals of H_2_pia and the four H-5 signals of two Hdpa ligands in 1·(OTf)_2_·H_2_O (indicated by inverted triangles; 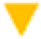, 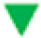, 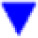, 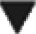) simultaneously shifted to a higher field, suggesting that additional F^−^ anions interacted with all three ligands and could not distinguish between the NH protons in Hdpa and amide protons in H_2_pia in 1·(OTf)_2_·H_2_O. This is consistent with the spectrophotometric titration results featuring the isosbestic points.

Among the signals of the E-ring (H_2_pia), the H-4e (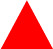) and H-6e (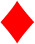) signals shifted to a higher field, whereas the H-3e (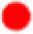) signal shifted to a lower field. These conflicting shifts are similar to those of the first step in the two-step reaction of [Ru(bpy)_2_(H_2_pia)](OTf)_2_ (P2·(OTf)_2_) with F^−^.^30^ In the case of the reaction between P2·(OTf)_2_ and F^−^ anions, the H-3e signal shifted to a lower field until an equimolar amount of TBAF for the complex was added; moreover, the H-3e signal shifted to a higher field when one-to-two equimolar amounts of TBAF were added (Fig. 7 and S19a†). This suggests that for P2·(OTf)_2_, the first F^−^ interacted with the proton of the amide group and the H-3e proton, and the second F^−^ linked to another proton of the amide. In contrast, the H-3e signal in the 1·(OTf)_2_·H_2_O complex shifted only to the lower field, suggesting that only the first F^−^ interacted with both the proton of the amide and the H-3e proton of H_2_pia in 1^2+^; however, the second F^−^ did not follow.

**Fig. 8:**
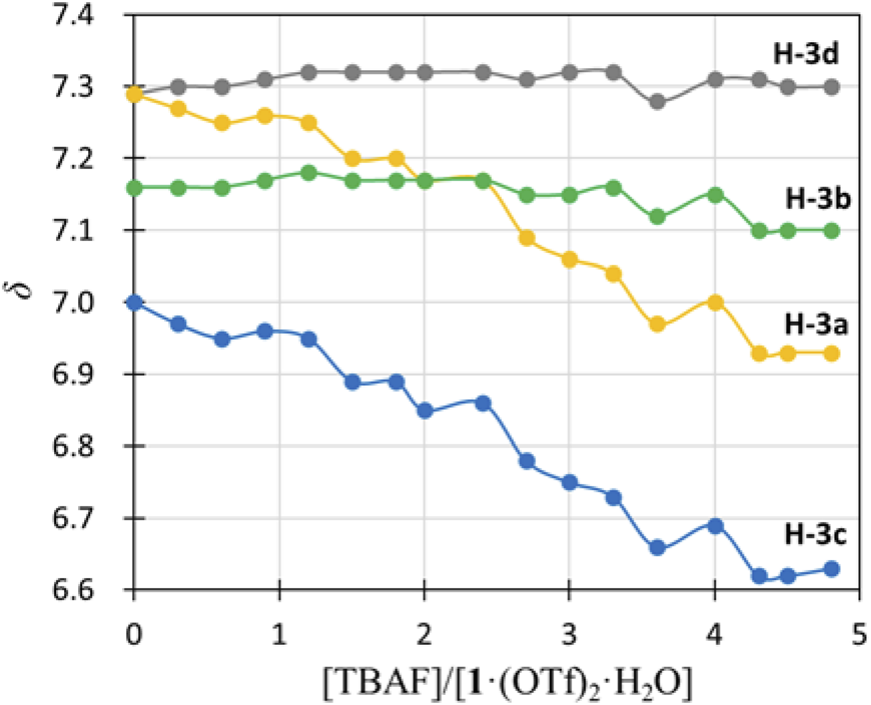
Titration profiles in terms of chemical shifts of the H-3 signal of the Hdpa ligands in 1·(OTf)_2_·H_2_O in DMSO-*d*_6_*vs.* [TBAF]/[1·(OTf)_2_·H_2_O].

The four H-5 signals corresponding to the two Hdpa ligands in 1·(OTf)_2_·H_2_O (indicated by inverted triangles; 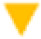, 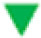, 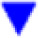, 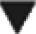) shifted to a higher field with increasing amount of TBAF added (Fig. 7 and S20†), indicating that each NH proton of the two Hdpa ligands established links with the F^−^ anions. With respect to the H-3 signals, the H-3a (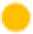) and H-3c (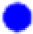) signals also shifted to a higher field; however, H-3b (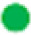) and H-3d (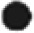) almost remained at the same chemical shifts (Fig. 7 and 8); that is, the H-3 signals corresponding to the Hdpa of the A- and C-rings shifted, whereas those corresponding to the Hdpa of the B- and D-rings were not. This suggests that an F^−^ anion that interacted with an Hdpa ligand was connected to an NH proton and one of the two H-3 protons in the Hdpa ligand to form the N^−^⋯H–F⋯H-3 hydrogen bonds. The shift of the H-3e signal of H_2_pia to a lower field was due to the chelating hydrogen-bonding interactions of F^−^. Therefore, the lack of change in the chemical shifts of H-3b and H-3d was due to the electron-donating effect of the F-adduct (N^−^⋯H–F) balancing the electron-attracting effect of the hydrogen bond (H–F⋯H-3). In the ^1^H NMR spectrum of 1·Cl_2_·2.5H_2_O, an NH proton and both H-3 protons in the two pyridyl groups of the Hdpa ligand formed hydrogen bonds with a Cl^−^ anion, and showed signals that were shifted to a lower field. The electron-donating effect of the Cl^−^ adduct (N–H⋯Cl^−^) was diminished compared to the effect of the hydrogen bond (H–F⋯H-3), and the H-3 signals shifted to lower values. Cl^−^ did not distinguish between the two H-3 protons of the Hdpa ligand (Fig. 4). However, the course of the reaction between 1·(OTf)_2_·H_2_O and F^−^ anions indicated that the F^−^ anion distinguished the two H-3 protons in the Hdpa ligand and favored the H-3b and H-3d protons over the H-3a and H-3c protons, respectively.

**Fig. 9:**
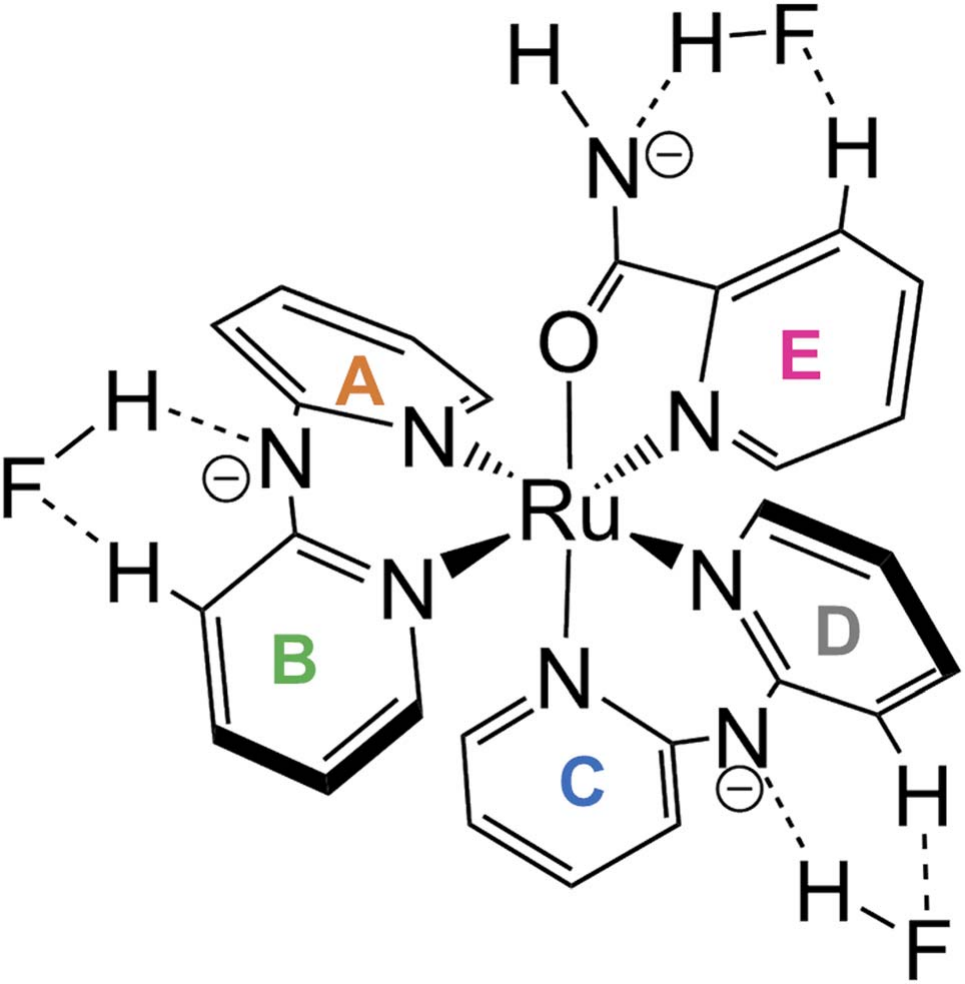
Proposed structure of tris-F-adduct-1^2+^, 1·F_3_^−^.

Thus far, the interactions at three sites—an amide of H_2_pia and two NH of Hdpa—have been discussed separately. Surprisingly, these interactions were found to proceed simultaneously, in contrast to our initial prediction (stepwise interactions). The changes in the absorption spectra suggest that one fluoride adduct was formed in a single step, and the variations in the NMR spectra suggest that the three fluoride-accepting sites (NH and NH_2_ groups) interacted simultaneously. Therefore, the reaction of 1^2+^ with F^−^ anions in DMSO was believed to form tris-F-adduct-1^2+^, 1·F_3_^−^ (Fig. 9). The proposed structure shows interactions of the H_2_pia ligand *via* the two H atoms of NH and H-3 with an F^−^ anion, similar to the F1 atom in the crystal structure of the fluoride salt. Similarly, each Hdpa ligand also interacted *via* the two H atoms of NH and H-3 with its respective F^−^ anion. These interactions were also observed in the crystal structure in which the Hdpa ligands with hydrogen-bonding interactions (NH–F2) were also hydrogen bonded to F2 *via* H-3 of one pyridyl group (Section 3.2). H-3 in the crystal structure, which acts as a hydrogen-bond donor to F2, corresponds to the H-3b and H-3d protons in the ^1^H NMR study; thus, the proposed structure (Fig. 9) is an exact mimic of the crystal structure of 1·F_2_·2EtOH.

### 3.6 Absorption spectra and DFT calculations

DFT calculations were performed at the B3LYP/LANL2DZ/6-31G* level in vacuum using the Spartan’20 program.^36^ The calculated structure of 1^2+^ (Table S4†) was consistent with the crystallographic data; the differences in the bond lengths and angles were within 0.073 Å and 1.8°, respectively (Table S5†). The Ru1–N4 bond, which is *trans* to the coordinated O atom of H_2_pia, was shorter than the five Ru–N bonds. The *trans* influence of the O atom of H_2_pia was observed in both structures of 1^2+^ (exp. and calc.).

In the calculated structure of the tris-F-adduct-1^2+^, 1·F_3_^−^ (Table S6†), each of the three fluoride ions was bound to a different NH group with F–H and F–N lengths of 1.01–1.00 and 2.529–2.545 Å, respectively, with the N–H bond lengths being elongated to 1.52–1.55 Å. The calculated structure of 1·F_3_^−^ is consistent with the crystallographic data; the differences in the bond lengths and angles were within 0.06 Å and 3.2°, respectively (Table S5†). With respect to the N–F distances in the calculated structure, those of the Hdpa site were shorter than the crystallographic counterparts by 0.05 Å, and that of the H_2_pia site was shorter than the crystallographic data by 0.149 Å. Unfortunately, the Hdpa ligands were hydrogen bonded to both the H-3 atoms of the two pyridyl groups, so that the added F^−^ anion approached not just one pyridine ring of the Hdpa ligands, as shown in the proposed structure shown in Fig. 9. However, the interactions between the complex cation 1^2+^ and fluoride ions were adequately calculated; however, the considerable difference in the interactions of the H_2_pia site was probably due to the solvation of fluoride ions and the crystal packing force. The two Hdpa ligands in the calculated structure had a flatter conformation than that of the counterparts in the calculated structure of 1^2+^. The dihedral angle of the calculated conformation of Hdpa(*N*4,*N*6) is consistent with the flat conformation indicated by the crystallographic data of 1·F_2_·2EtOH within 0.35° (Table S5†). The calculated flat conformation of Hdpa(*N*1,*N*3) did not adequately match the bent conformation of Hdpa(*N*1,*N*3) in the crystal, which was likely deformed by the crystal packing force.

**Fig. 10:**
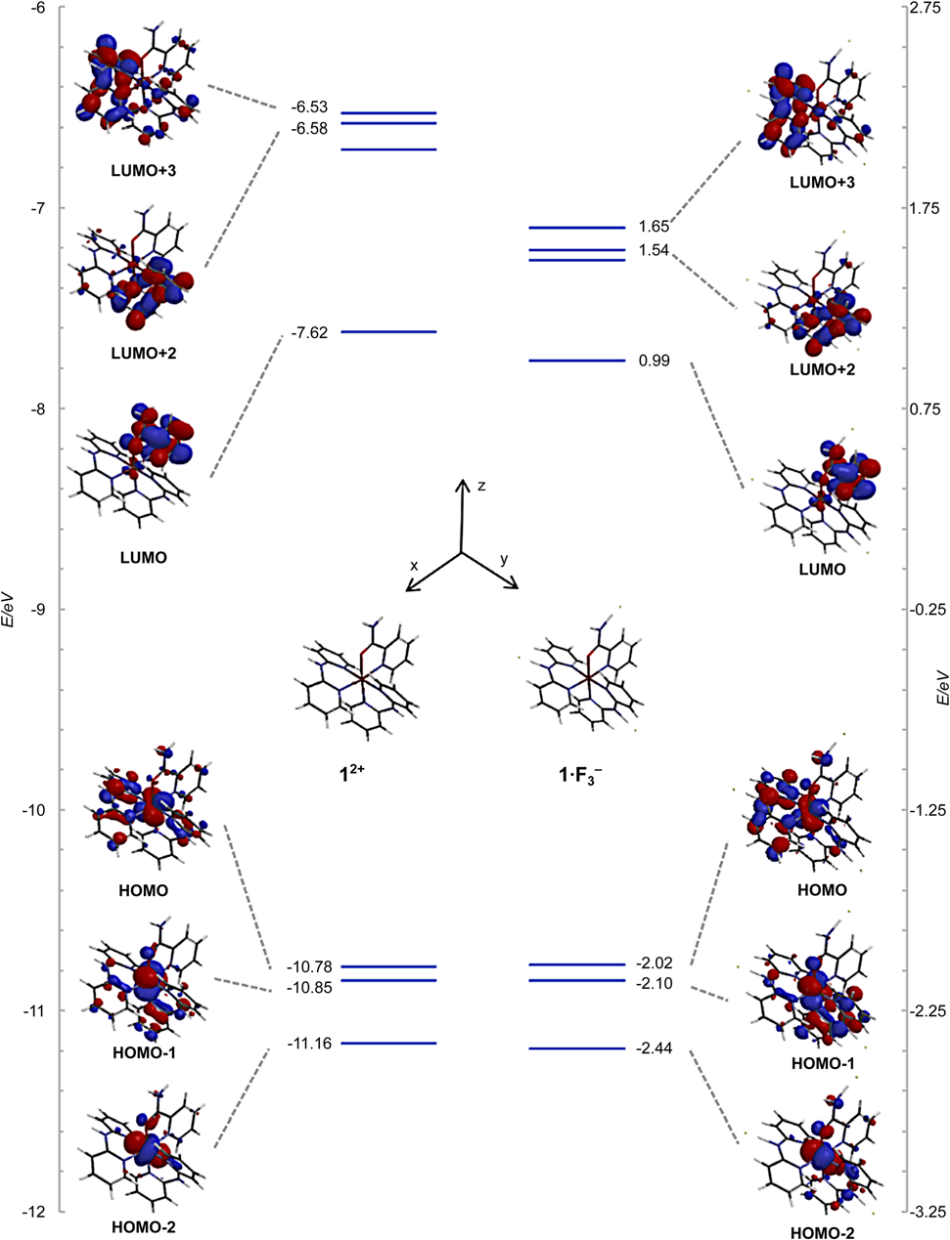
Energy-level diagrams and frontier orbitals of 1^2+^ and 1·F_3_^−^ calculated at the B3LYP/LANL2DZ/6-31G* level in vacuum.

The frontier orbitals and energy levels of 1^2+^ and 1·F_3_^−^ are shown in Fig. 10, in which the vertical energy axis was adjusted such that the highest occupied molecular orbital (HOMO)−1 positions were at the same level. The HOMOs of 1^2+^ and 1·F_3_^−^ were attributed to the Ru(d_*xy*_ and d_*yz*_) and Hdpa(*N*1,*N*3)(n and π) MOs, which featured contributions from the non-bonding MOs in the bridged N atom and *π* MOs of the two pyridyl groups. The HOMO−1 levels in both species were ascribed to the Ru(d_*xz*_) and Hdpa(*N*4,*N*6)(n and π) MOs, whereas the HOMO−2 levels corresponded to the Ru(d_*xy*_ and d_*yz*_) MOs. The energy levels of the HOMOs, HOMOs-1, and HOMOs-2 were not affected by the fluorine-ion adduct. The lowest unoccupied molecular orbitals (LUMOs) and LUMOs+1 of both species corresponded to H_2_pia(π*) orbitals. The LUMOs+2 and LUMOs+3 positions of both species were ascribed to Hdpa(*N*4,*N*6)(π*) and Hdpa(*N*1,*N*3)(π*) orbitals, respectively. The HOMO–LUMO gap of 1·F_3_^−^ was smaller than that of 1^2+^, and the gaps of HOMO–LUMOs+2 or +3 in 1·F_3_^−^ were also smaller than that of 1^2+^, with the decrease being greater than that of HOMO–LUMO. Thus, the stabilization of the LUMOs of Hdpa(π*) was attributed in part to the extension of the π system due to the flatter conformation of the Hdpa ligands.

**Fig. 11:**
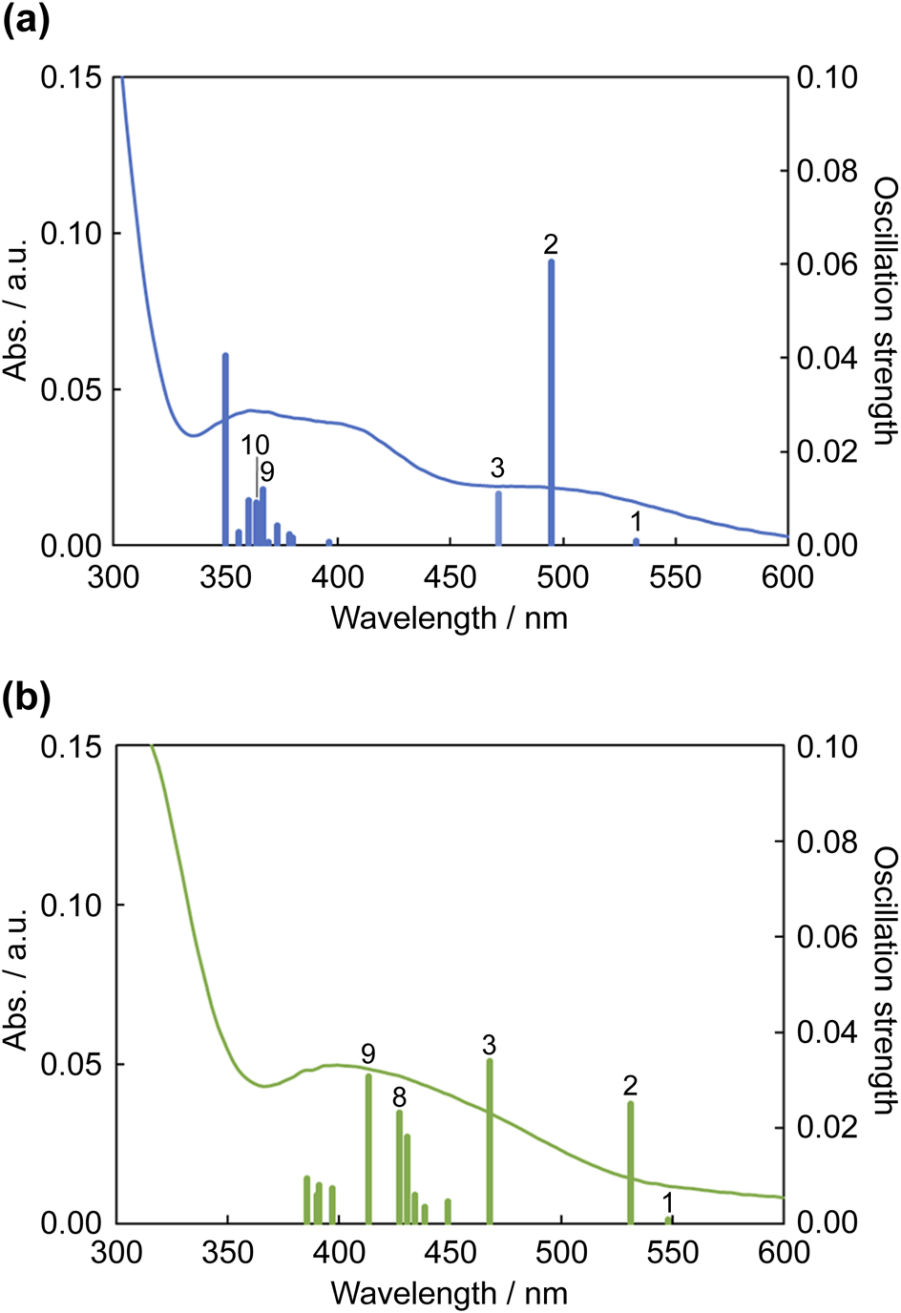
Calculated electronic absorption spectra of (a) 1^2+^ (a) and (b) 1·F_3_^−^. Solid curves represent the electronic absorption spectra collected using DMSO.

The results of TDDFT calculations are summarized in Fig. 11 and Table 3. The absorption band at ∼500 nm for 1^2+^ was assigned to the HOMO → LUMO transition; moreover, the S2 transition energy for 1^2+^ was calculated to be 495 nm. The S2 transition for 1·F_3_^−^ was red-shifted to 532 nm, and its oscillation strength was reduced by approximately half. The S3 transition energies for 1^2+^ and 1·F_3_^−^ were similar; however, the S3 oscillation strength for 1·F_3_^−^ was approximately three times greater than that for 1^2+^. Therefore, the increase in S3 oscillation strength induced an absorption band at ∼450 nm for 1·F_3_^−^. The MLCT to Hdpa(π*) of 1^2+^ was achieved by the S9 and S10 transitions at 367 and 364 nm, respectively. For the MLCT to Hdpa(π*) in 1·F_3_^−^, the S8 and S9 transitions were red-shifted to 427 and 413 nm, respectively, which also contributed to the absorption band at ∼450 nm.

**Table 3:** Selected transitions of time-dependent density functional theory (TDDFT) calculations of 1^2+^ and 1·F_3_^−^

	Sn	/nm	*F* a	Dominant transition (percentage contribution)^b^	
1^2+^	10	363.82	0.0091	HOMO → LUMO+1	(34%)
				HOMO → LUMO+2	(23%)
				HOMO → LUMO+3	(13%)
	9	366.66	0.0119	HOMO → LUMO+2	(45%)
				HOMO → LUMO+1	(16%)
				HOMO-1 → LUMO+1	(16%)
				HOMO-1 → LUMO+2	(10%)
	3	471.21	0.0109	HOMO-2 → LUMO	(93%)
	2	494.78	0.0604	HOMO → LUMO	(90%)
	1	532.58	0.0009	HOMO-1 → LUMO	(96%)
1·F_3_^−^	9	413.43	0.0308	HOMO-1 → LUMO+3	(79%)
	8	427.22	0.0231	HOMO-1 → LUMO+2	(60%)
				HOMO → LUMO+2	(14%)
	3	468.07	0.0339	HOMO-2 → LUMO	(82%)
	2	531.56	0.0249	HOMO → LUMO	(85%)
	1	548.22	0.0008	HOMO-1 → LUMO	(95%)

a Oscillator strength.

b Actual percent contribution = (configuration coefficient)^2^ × 2 × 100%.

## 4 Conclusions

A bis-heteroleptic Ru(ii) complex with Hdpa and H_2_pia ligands, [Ru(Hdpa)_2_(H_2_pia)]Cl_2_·2.5H_2_O (1·Cl_2_·2.5H_2_O) was selectively obtained by reacting *cis*-[RuCl(dmso-*S*)(Hdpa)_2_]Cl (P1·Cl) with the H_2_pia ligand (yield 91%). A triflate salt 1·(OTf)_2_·H_2_O was quantitatively prepared by adding a suitable amount of Li(OTf) to an aqueous solution of 1·Cl_2_·2.5H_2_O. Similarly, a fluoride salt 1·F_2_·4H_2_O was obtained by adding a suitable amount of TBAF·3H_2_O to an acetonitrile solution of 1·(OTf)_2_·H_2_O.

The crystal structures of 1·Cl_2_·2.5H_2_O and 1·F_2_·2EtOH revealed almost identical geometries of the 1^2+^ dication. The H_2_pia ligand in 1^2+^ was coordinated to the Ru(ii) ion *via* a pyridyl-N and carbonyl-O atom of the amide group, and the π-electron on the carbonyl was delocalized over the entire amide group. Similar structural features have also been observed for [Ru(bpy)_2_(H_2_pia)]^2+^ (P2^2+^). The two Hdpa ligands in 1·Cl_2_·2.5H_2_O exhibited a general bent conformation of the coordinated Hdpa ligand, and both NH groups in each Hdpa ligand pointed in opposite directions (*anti*-NH conformation). In 1·F_2_·2EtOH, the Hdpa ligands were also bent in an interdigitated conformation; however, one of them had an unusually smaller dihedral angle (15.8°) than the others (29.9°–35.0°) owing to the F^−^ anion, which hydrogen bonded with the NH group in the Hdpa ligands. The crystals were found to contain hydrogen-bonding networks.

In the chloride salt, two Cl^−^ ions exhibited hydrogen-bonding interactions with each NH group of the two Hdpa ligands, and two 1^2+^ complexes were connected with two Cl_2_ atoms to form a dimer structure. These dimers were observed along the *a*-axis. In the fluoride salt, an F^−^ ion connects two 1^2+^ cations *via* two Hdpa ligands to form 1D hydrogen-bonding networks. Moreover, another F^−^ ion also connects two 1^2+^ cations *via* hydrogen-bonding interactions that included ethanol molecules. Consequently, the 1D hydrogen-bonding chains with F2 atoms are crosslinked *via* hexagonal hydrogen bonding, including F1 atoms, to form a 3D network. Therefore, the F^−^ ion plays a different role in the crystal from that of a Cl^−^ ion.

Upon the addition of TBAF to the red DMSO solution of 1·(OTf)_2_·H_2_O, the solution turned dark brown. The reaction was as expected because P1·(OTf) and P2·(OTf)_2_ were also capable of achieving naked-eye fluoride detection. ^1^H NMR and absorption spectroscopy of the reaction between 1^2+^ and additional F^−^ anions revealed that the additional F^−^ anion could not distinguish between the NH groups of the Hdpa ligands and the amide group in the H_2_pia ligand, although they were in different environments in the DMSO solution. The presence of sufficient F^−^ anions in the solution led to the formation of tris-F-adduct 1·F_3_^−^. The amide group of H_2_pia in 1^2+^ could accept only one F^−^ anion to accommodate the H-3 proton in the pyridyl group and amide proton in H_2_pia. Similarly, other F^−^ anions were accommodated between the H-3 proton in the pyridyl group and the NH proton in Hdpa in 1^2+^. The three interactions proceeded simultaneously at the three sites.

Moreover, the presence of Cl^−^ anions in the solution did not obstruct the reaction between 1^2+^ and F^−^ anions, although Cl^−^ anions formed hydrogen bonds with the amino and amide protons in 1^2+^ in solution. The F^−^ anion established stronger contact with 1^2+^ than the Cl^−^ anion. The masking effect of Cl^−^ anions was not observed in our previous study. Based on the absorption spectroscopic data, the detection limits for F^−^ in DMSO were calculated to be ∼0.68 × 10^−5^ M and ∼0.89 × 10^−5^ M for 1·(OTf)_2_·H_2_O and 1·Cl_2_·2.5H_2_O, respectively.

The DFT-calculated structures of 1^2+^ and tris-F-adduct 1·F_3_^−^ were consistent with the crystallographic data; the corresponding differences in the bond length were within 0.073 and 0.06 Å, and those in the bond angle were within 1.8 and 3.2°, respectively. The HOMO–LUMOs+2 or +3 gap of 1·F_3_^−^ was smaller than that of 1^2+^, and the decrease was greater than that of HOMO–LUMO. Thus, the stabilization of the LUMOs of Hdpa(π*) was attributed in part to the extension of the π system due to the flatter conformation of the Hdpa ligands. TDDFT calculations of 1^2+^ and tris-F-adduct 1·F_3_^−^ were consistent with their absorption spectra.

## Author contributions

M. T. conceived and directed the research, analyzed all the data, and wrote the manuscript with input from all authors. T. H. prepared and characterized the compounds. N. N. collected, solved, and refined the crystal structures in addition to performing and analyzing the quantum chemical calculations.

## Conflicts of interest

There are no conflicts of interest to declare.

## Supplementary Material

RA-012-D2RA03593F-s001

RA-012-D2RA03593F-s002
