# Supplementary material for: Colorimetric fluoride detection in dimethyl sulfoxide using a heteroleptic ruthenium(ii) complex with amino and amide groups: X-ray crystallographic and spectroscopic analyses
Source: RSC Adv. 2022 Sep 7;12(39):25227–39. doi: 10.1039/d2ra03593f (PMC9450000; doi:10.1039/d2ra03593f)
Supplement: RA-012-D2RA03593F-s001 [file RA-012-D2RA03593F-s001.pdf]

Electronic Supplementary Material (ESI) for RSC Advances.
This journal is © The Royal Society of Chemistry 2022

Electronic Supplementary Information

Colorimetric fluoride detection in dimethyl sulfoxide solution using a heteroleptic ruthenium(II) complex with amino and amide groups: X-ray crystallographic and spectroscopic analyses

Mari Toyama, Tomoki Hasegawa, and Noriharu Nagao

Table S1. Hydrogen bonds and related parameters (Å, deg) for **1**·Cl_2·2.5H_2O and **1**·F_2·2EtOH

D-H···A	D-H	H···A	D···A	D-H···A
Chloride salt				
N2-H···Cl2	0.86	2.35	3.21	174.9
N5-H···Cl1	0.87	2.33	3.17	163.5
N8-H8A···Cl2*	0.88	2.54	3.38	158.8
Fluoride salt				
N2-H···F2	0.85	1.74	2.59	171.0
N5-H···F2^1	0.84	1.74	2.58	171.7
N8-HB···F1	0.86	1.77	2.62	173.8
O2-H···F1	0.84	1.72	2.56	177.4
O3-H···F1	0.91	1.72	2.57	152.4
N8-HA···O2^2	0.86	2.08	2.89	156.4

(*) -x, -y, -z, (1) 1+x, 1.5-y, 1/2+z, (2) -x, 2-y, z

Table S2. Sets of ^{1}H NMR signals on five pyridyl rings of two Hdpa and an H_2pia ligands.

(a) **1**·$(OTf)_2$·H_2O

**Set**	**H-3**	**H-4**	**H-5**	**H-6**	**Ring assigned**
**1**	7.00	7.65	6.89	7.54	**C**
**2**	7.17	7.73	6.73	7.62	**B**
**3**	7.30	7.86	6.81	7.60	**A**
**4**	7.30	7.85	6.82	7.81	**D**
**5**	8.19	8.06	7.81	8.94	**E**

(b) **1**·Cl_2·2.5H_2O

**Set**	**H-3**	**H-4**	**H-5**	**H-6**	**Ring assigned**
**1**	7.36	7.63	6.87	7.55	**C**
**2**	7.55	7.70	6.70	7.60	**B**
**3**	7.65	7.82	6.78	7.55	**A**
**4**	7.72	7.82	6.78	7.82	**D**
**5**	8.42	8.04	7.82	8.91	**E**

Table S3. Chemical shifts of ^{1}H NMR spectra of **1**·$(OTf)_2$·H_2O and **1**·Cl_2·2.5H_2O in DMSO-d_6

	Triflate salt	Chloride salt	$\Delta(\delta_{Cl} - \delta_{(OTf)})$
NH(Hdpa)	10.74	11.85	1.11
NH(Hdpa)	10.62	11.52	0.90
NH (H_2pia)	9.68	9.81	0.13
NH' (H_2pia)	9.50	9.79	0.29
pyridyl-rings of Hdpa			
H-3a	7.30 (in multiplet)	7.65	0.35
H-3b	7.17	7.55 (in multiplet)	0.38
H-3c	7.00	7.36	0.36
H-3d	7.30 (in multiplet)	7.72	0.42
H-4a	7.86	7.82 (in multiplet)	-0.04
H-4b	7.73	7.70	-0.03
H-4c	7.65	7.63	-0.02
H-4d	7.85	7.82 (in multiplet)	-0.03
H-5a	6.81	6.78 (in multiplet)	-0.03
H-5b	6.73	6.70	-0.03
H-5c	6.89	6.87	-0.02
H-5d	6.82	6.78 (in multiplet)	-0.04
H-6a	7.60	7.55 (in multiplet)	-0.05
H-6b	7.62	7.60	-0.02
H-6c	7.54	7.55 (in multiplet)	0.01
H-6d	7.81 (in multiplet)	7.82 (in multiplet)	0.01
pyridyl-ring of H_2pia			
H-3e	8.19	8.42	0.23
H-4e	8.06	8.04	-0.02
H-5e	7.81	7.82 (in multiplet)	-0.01
H-6e (in multiplet)	8.94 (in multiplet)	8.91	-0.03

Table S4. DFT optimized coordinates (Å) of **1**$^{2+}$ calculated using Spartan'20 at the B3LYP/LANL2DZ/6-31G* level in vacuum. The final energy is −1612.05570597404 Hartree

ATOM	X/Å	Y/Å	Z/Å
1 Ru	-0.055910347	-0.034334723	-0.166446773
2 N	-0.502667242	0.067347745	1.892728071
3 C	-1.000506676	0.079313101	4.653713621
4 C	-1.091998587	-0.987291534	2.517127634
5 C	-0.221453194	1.158117334	2.640704833
6 C	-0.443687876	1.182332792	4.028538013
7 C	-1.348690834	-1.029364524	3.874626212
8 H	-1.344871224	-1.819430677	1.874196805
9 H	-0.198879994	2.075675534	4.595403281
10 H	-1.811360936	-1.908093407	4.309499334
11 H	-1.18200079	0.090725325	5.723675256
12 N	-0.195947538	2.083938394	-0.254016536
13 C	-0.184458035	4.866961536	-0.519008356
14 C	0.065015895	2.871052604	0.806803804
15 C	-0.491083742	2.681832395	-1.436664376
16 C	-0.494211015	4.053072664	-1.614692264
17 C	0.089842468	4.272217195	0.70182971
18 H	-0.715064391	2.003785295	-2.250948847
19 H	-0.734081425	4.473395342	-2.584839527
20 H	0.30254193	4.876031765	1.578942455
21 H	-0.175627362	5.948271834	-0.613030711
22 N	-2.123235558	-0.263129381	-0.602708999
23 C	-4.77098669	-0.331294293	-1.512145798
24 C	-3.035965288	0.659377279	-0.203770791
25 C	-2.548540203	-1.261080858	-1.405356845
26 C	-3.865982563	-1.306501066	-1.894431267
27 C	-4.351340391	0.668577942	-0.627599141
28 H	-2.662911176	1.419469449	0.470143456
29 H	-4.168642815	-2.119145324	-2.547992096
30 H	-5.028060638	1.439871533	-0.277814013
31 H	-5.792322529	-0.360651765	-1.878260365

Table S4. (continued)

ATOM		X/Å	Y/Å	Z/Å
32	N	0.087197615	-2.166920315	-0.19890714
33	C	0.586341073	-4.896693488	-0.564812438
34	C	-0.651807428	-2.888558809	-1.066535389
35	C	1.031795438	-2.819579637	0.525886165
36	C	1.316855653	-4.16349655	0.37946618
37	C	-0.408672265	-4.258054416	-1.283413313
38	H	1.572002542	-2.20775147	1.238523504
39	H	2.089363954	-4.62473579	0.984290603
40	H	-1.019291739	-4.805581417	-1.995336425
41	H	0.778149035	-5.953883407	-0.718710375
42	N	0.348809553	2.291684105	2.048980317
43	H	0.602982339	2.987850032	2.739364941
44	N	-1.67494234	-2.280767756	-1.789622919
45	H	-2.08736271	-2.903500323	-2.472897118
46	N	2.073614851	0.1597171	-0.101120012
47	C	4.856077307	0.439638683	-0.304641025
48	C	2.841806755	0.291865319	0.993629528
49	C	2.688644289	0.154757252	-1.31765601
50	C	4.069682931	0.295638063	-1.447477505
51	C	4.227240591	0.433334486	0.937572146
52	H	2.31830042	0.284279046	1.941552505
53	H	4.54406505	0.297024143	-2.422913879
54	H	4.793765206	0.537508609	1.856964886
55	H	5.932136106	0.552241113	-0.387241697
56	C	1.743791521	-0.010154947	-2.46224914
57	O	0.51304156	-0.103925226	-2.232366711
58	N	2.200453024	-0.051605764	-3.7196745
59	H	3.176462497	0.018302024	-3.965460081
60	H	1.532585932	-0.159680163	-4.47340088

Table S5. Selected bond lengths (Å), angles (deg), and dihedral angles (deg) for **1**·Cl_2·2.5H_2O (that is, $\mathbf{1}^{2+}$(exp.)), $\mathbf{1}^{2+}$(calc.), **1**·F_2·2EtOH(exp.), and **1**·F_3^-(calc.)

	$\mathbf{1}^{2+}$(exp.)	$\mathbf{1}^{2+}$(calc.)	**1**·F_2·2EtOH(exp.)	**1**·F_3^-(calc.)
Ru1-N1	2.078(6)	2.137	2.0861(15)	2.134
Ru1-N3	2.062(6)	2.125	2.0705(15)	2.118
Ru1-N4	2.049(5)	2.109	2.0517(14)	2.111
Ru1-N6	2.073(5)	2.125	2.0693(15)	2.116
Ru1-N7	2.066(6)	2.139	2.0621(15)	2.112
Ru1-O1	2.099(5)	2.144	2.1058(12)	2.134
O1-C26	1.268(9)	1.256	1.269(2)	1.289
C26-N8	1.324(10)	1.338	1.304(2)	1.307
C21-C26	1.485(10)	1.493	1.496(2)	1.509
N1-Ru1-N3	87.5(2)	87.42	87.71(6)	88.41
N4-Ru1-N6	89.4(2)	88.75	90.77(6)	89.75
N7-Ru1-O1	78.4(2)	76.61	78.16(5)	77.64
C1-N2-C6	128.0(6)	129.48	127.97(15)	129.94
C11-N5-C16	127.9(5)	128.86	131.75(16)	130.44
O1-C26-N8	120.9(7)	120.27	122.10(17)	125.26
O1-C26-C21	118.5(6)	119.22	117.63(16)	116.55
C21-C26-N8	120.5(7)	120.51	120.22(17)	118.19
Ru1-N1-C2-N2	10.1(9)	10.94	-20.1(2)	-15.80
Ru1-N3-C6-N2	-14.0(8)	-10.24	5.4(2)	12.67
Ru1-N4-C11-N5	-6.0(8)	-2.03	5.2(2)	6.65
Ru1-N6-C16-N5	4.1(9)	4.69	-10.7(2)	-8.55
Plane(1)-plane(2)	34.8	31.11[1)]	31.7	20.03[1)]
Plane(3)-Plane(4)	30.0	32.36[2)]	15.8	16.15[2)]
N2-F1	-	-	2.585	2.540
N5-F1	-	-	2.576	2.529
N7-F2	-	-	2.694	2.545

Plane(1) = N1, C1, C2, C3, C4, C5, Plane(2) = N3, C6, C7, C8, C9, C10,

Plane(3) = N4, C11, C12,C13,C14,C15, Plane(4) = N6, C16, C17, C18, C19, C20,

[1)] Plane(1) = N1, C2, C4, Plane(2) =N3, C7, C9,

[2)] Plane(3) = N4, C12, C14, Plane(4) =N6, C17, C19

Table S6. DFT optimized coordinates (Å) of **1**·F_3^- calculated using Spartan'20 at the B3LYP/LANL2DZ/6-31G* level in vacuum. The final energy is −1912.10923977040 Hartree

ATOM		X/Å	Y/Å	Z/Å
1	Ru	0.124993832	-0.116087638	-0.038617873
2	N	0.549899704	1.950066033	0.040950805
3	C	0.928267983	4.732429172	-0.030446637
4	C	0.937204938	2.593385413	-1.09053715
5	C	0.411739026	2.684778285	1.191685463
6	C	0.572145654	4.104323177	1.139525519
7	C	1.136719523	3.956086127	-1.184265109
8	H	1.072477977	1.951157251	-1.951501015
9	H	0.4234694	4.646620192	2.066826598
10	H	1.440849158	4.394374686	-2.129189224
11	H	1.059959086	5.811891188	-0.053234733
12	N	0.215149039	-0.206413903	2.073320271
13	C	0.068852946	-0.50555998	4.85262846
14	C	0.118875438	0.902547512	2.865189873
15	C	0.276014287	-1.428187811	2.658936493
16	C	0.212289196	-1.632130014	4.023985605
17	C	0.021586765	0.745562792	4.280918015
18	H	0.360690439	-2.255193306	1.96343357
19	H	0.263086714	-2.639725445	4.423325607
20	H	-0.06938351	1.650964746	4.870784099
21	H	0.003400373	-0.61418274	5.932952943
22	N	2.190269471	-0.542227283	-0.233262995
23	C	4.850178747	-1.427774443	-0.230689999
24	C	3.037501175	-0.252721191	0.784734904
25	C	2.676575828	-1.22534374	-1.314044935
26	C	4.021742008	-1.704986702	-1.292431462
27	C	4.355808317	-0.660220138	0.838707026
28	H	2.603464281	0.322645498	1.59324667
29	H	4.354562051	-2.264142127	-2.159983202
30	H	4.970826388	-0.394021186	1.692180588
31	H	5.87714324	-1.786439208	-0.230540338

Table S6. (continued)

ATOM		X/Å	Y/Å	Z/Å
32	N	-0.030040438	-0.190052006	-2.166138933
33	C	-0.583197393	-0.684801557	-4.870893199
34	C	0.82857133	-0.961519923	-2.901874907
35	C	-1.102408632	0.35748262	-2.789512115
36	C	-1.423758584	0.154618353	-4.116044095
37	C	0.526041225	-1.232737262	-4.273157057
38	H	-1.725557903	0.983642666	-2.161458078
39	H	-2.305092179	0.623924103	-4.541039879
40	H	1.225613339	-1.861959931	-4.812342353
41	H	-0.797522613	-0.889816375	-5.917368804
42	N	0.122565973	2.183502472	2.416565014
43	H	-0.07756618	3.241435748	3.51168612
44	N	1.991527731	-1.481778231	-2.453575392
45	H	2.734073633	-2.348437734	-3.46179897
46	N	-1.972654729	-0.0126844	0.188810772
47	C	-4.739763118	-0.156480948	0.506827088
48	C	-2.699718531	1.097601239	0.419594605
49	C	-2.611708414	-1.210988782	0.124288784
50	C	-3.997797812	-1.310951331	0.272833989
51	C	-4.078875154	1.069815819	0.585994269
52	H	-2.140392484	2.024623327	0.46738062
53	H	-4.468748364	-2.286342237	0.200182453
54	H	-4.614251776	1.995806635	0.773327572
55	H	-5.818040486	-0.216178636	0.628159673
56	C	-1.71394006	-2.402453726	-0.099699999
57	O	-0.447204646	-2.169842367	-0.138666416
58	N	-2.269297907	-3.579221652	-0.22487545
59	H	-3.735852554	-4.059127154	-0.149877014
60	H	-1.537059367	-4.27385439	-0.378115569
61	F	-4.71106314	-4.281087838	-0.069184388
62	F	-0.157568045	3.936434191	4.23318663
63	F	3.264327803	-2.850045912	-4.157802807

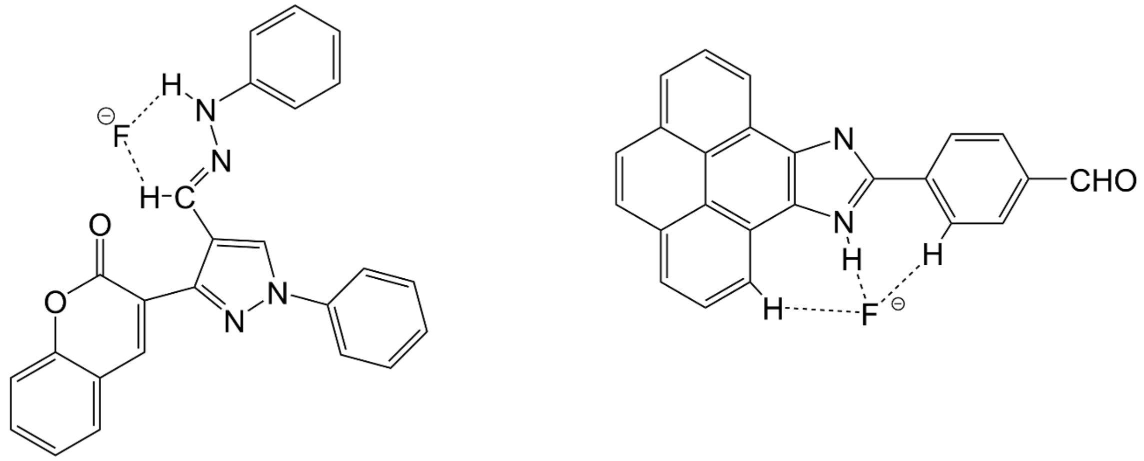

Fig. S1. Proposal structures of anion-receptors with an F^- anion. Ref. 16, 17.

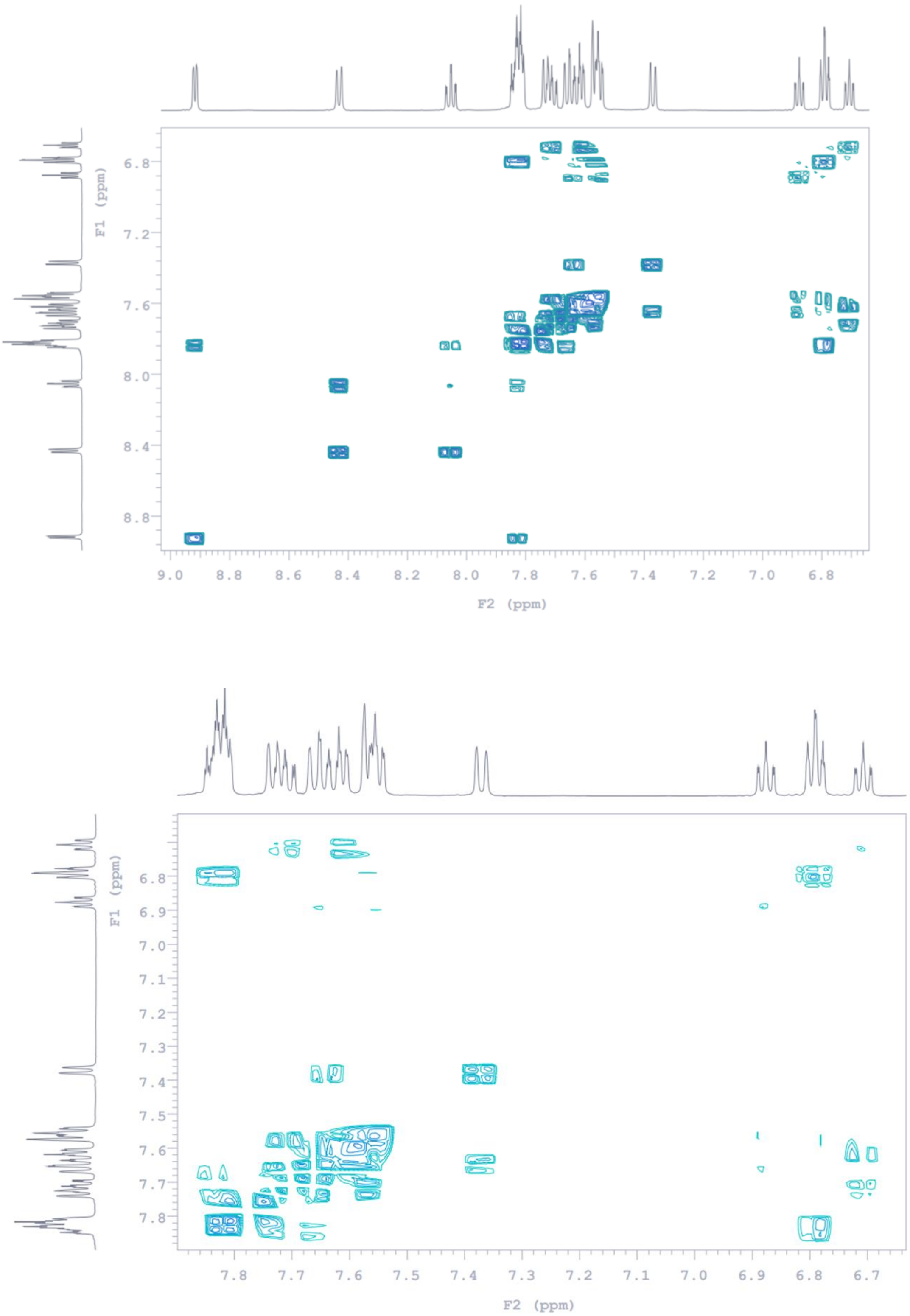

Fig. S2. ^{1}H-^{1}H COSY spectra of **1**$\cdot Cl_2 \cdot 2.5H_2O$ in DMSO-d_6 (500 MHz, 298 K).

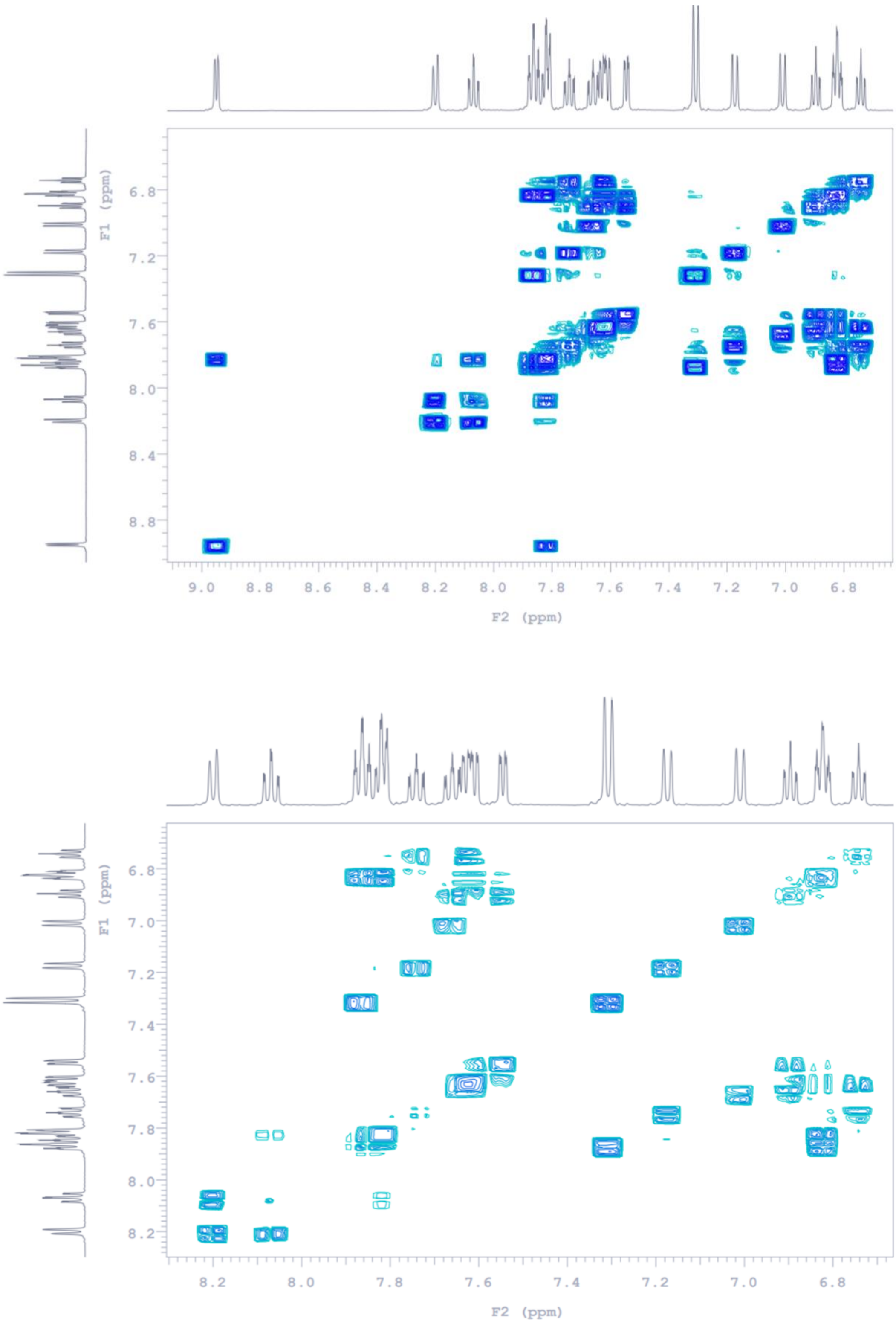

Fig. S3. ^{1}H-^{1}H COSY spectra of **1**·$(OTf)_2$·H_2O in DMSO-d_6 (500 MHz, 298 K).

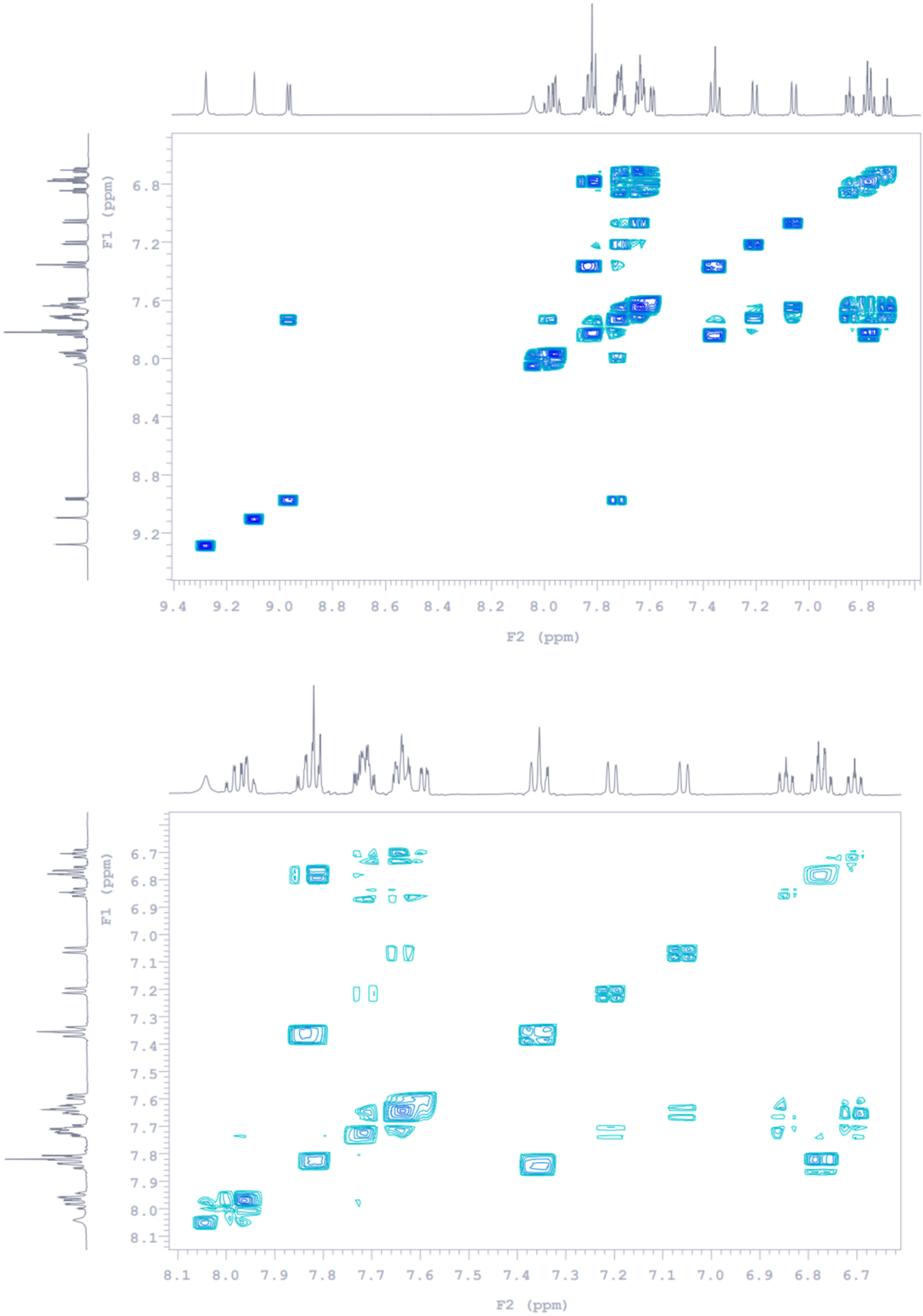

Fig. S4. ^{1}H-^{1}H COSY spectra of **1**·$(OTf)_2$·H_2O in CD_3CN (500 MHz, 298 K).

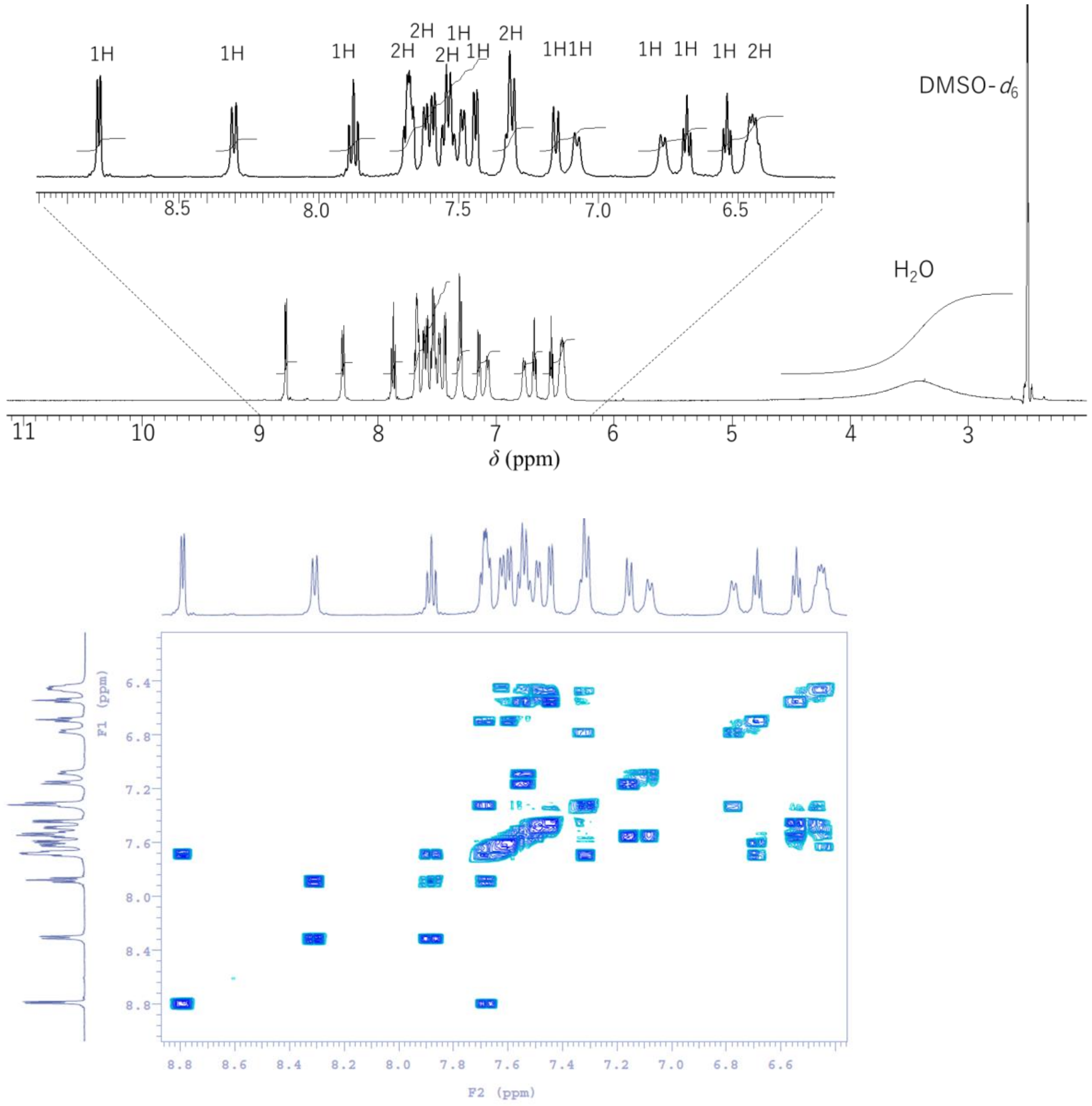

^{1}H NMR (500 MHz DMSO-d_6): δ = 6.44 (*m*, 2H, H-5a and H-5b), 6.53 (*dd*, 1H, ^{3}J = 6.0 and 5.0 Hz, H-5c), 6.68 (*dd*, 1H, ^{3}J = 6.0 and 7.0 Hz, H-5d), 6.76 (*d*, 1H, ^{3}J = 8.0 Hz, H-3a), 7.07 (*d*, 1H, ^{3}J = 8.0 Hz, H-3c), 7.15 (*d*, 1H, ^{3}J = 8.2 Hz, H-3b), 7.30 (*d*, 1H, ^{3}J = 8.0 Hz, H-3d), 7.31 (*dd*, 1H, ^{3}J = 6.0 and 8.0 Hz, H-4a), 7.43 (*d*, 1H, ^{3}J = 5.9 Hz, H-6c), 7.48 (*d*, 1H, ^{3}J = 5.7 Hz, H-6b), 7.53 (*m*, 2H, H-4b and H-4c), 7.59 (*d*, 1H, ^{3}J = 6.0 Hz, H-6a), 7.61 (*d*, 1H, ^{3}J = 6.0 Hz, H-6d), 7.67 (*m*, 2H, H-4d and H-5e), 7.87 (*dd*, 1H, ^{3}J = 7.7 and 7.9 Hz, H-4e), 8.30 (*d*, 1H, ^{3}J = 7.7 Hz, H-3e), 8.78 (*d*, 1H, ^{3}J = 5.0 Hz, H-6e).

Fig. S5. ^{1}H NMR and ^{1}H-^{1}H COSY spectra of **1**·F_2·$4H_2O$ in DMSO-d_6 (500 MHz, 298 K).

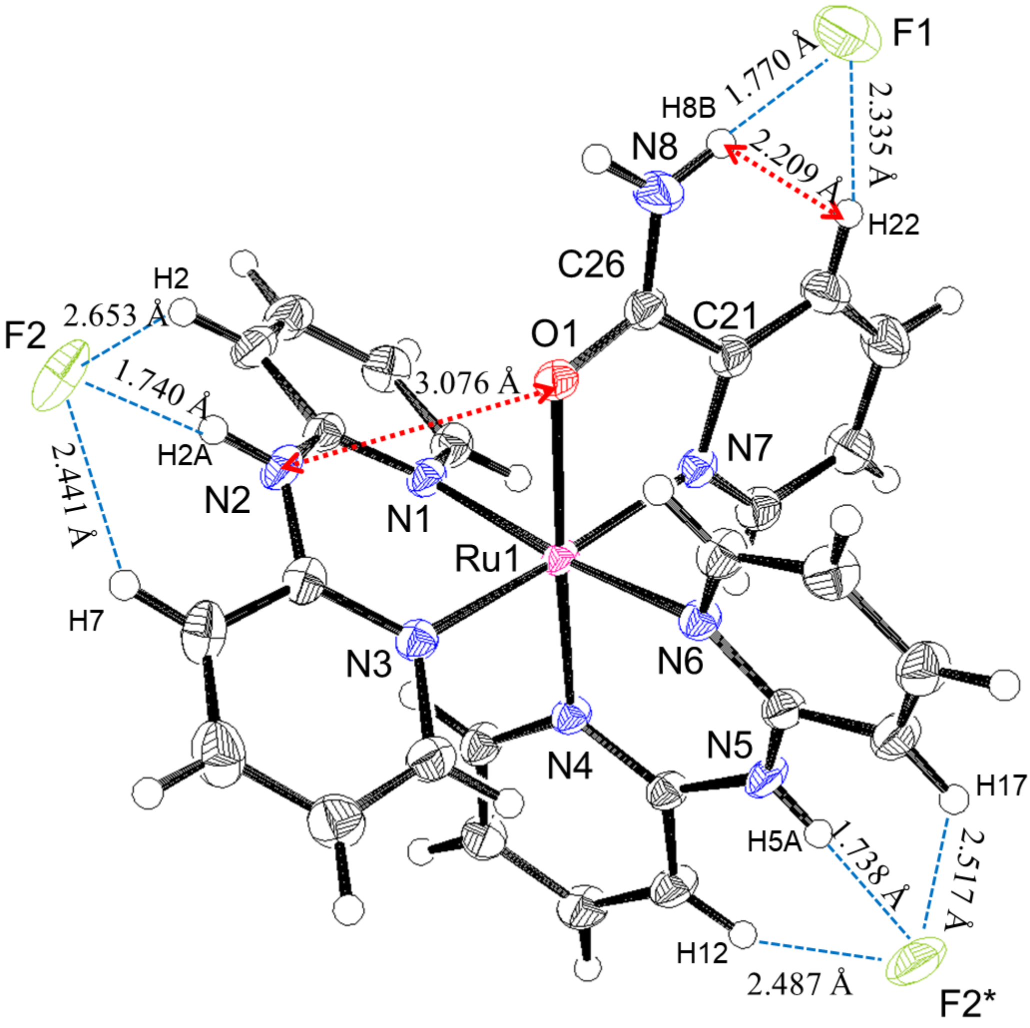

Fig. S6. ORTER drawing of **1**·F_2 in **1**·F_2·2EtOH. Ellipsoid are drawn at 50% probability level. Two ethanol molecules have been omitted for clarity. [(*) 1+x, 1.5-y, 1/2+z]

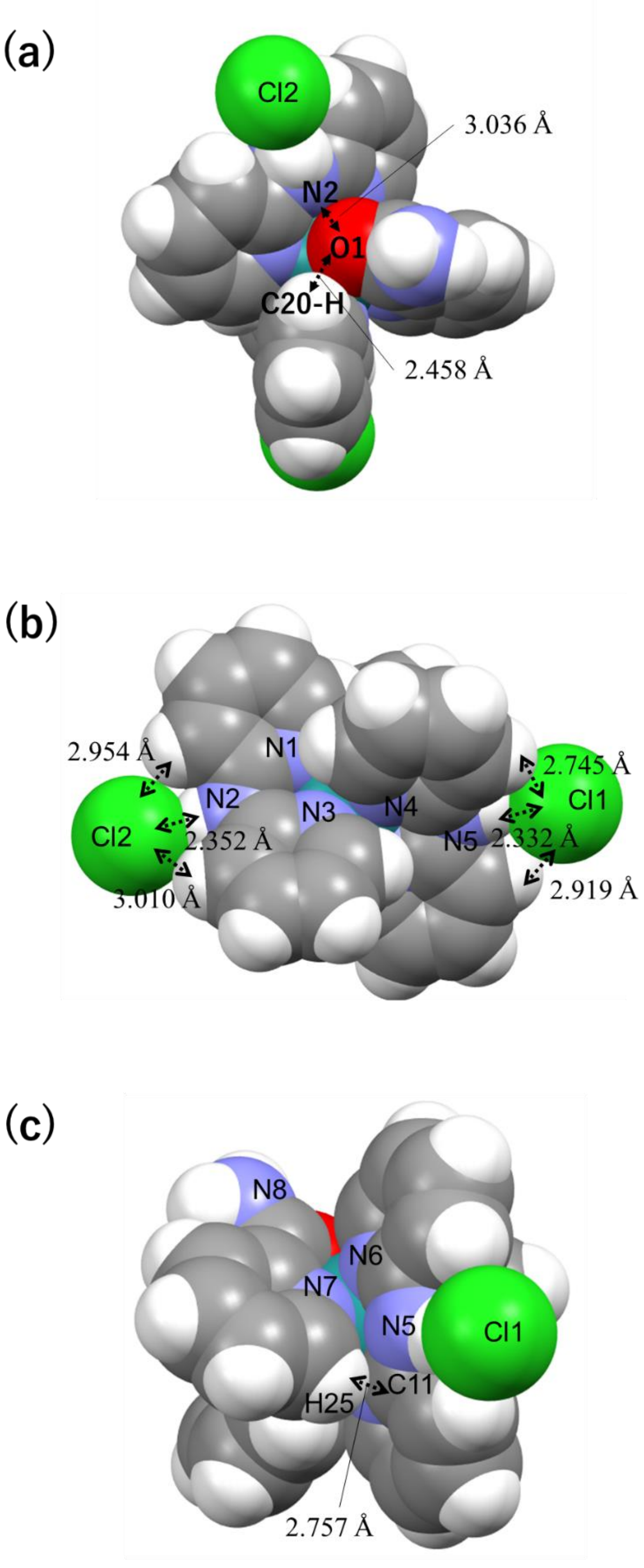

Fig. S7. Space Filling models of **1**·Cl_2.

(a)

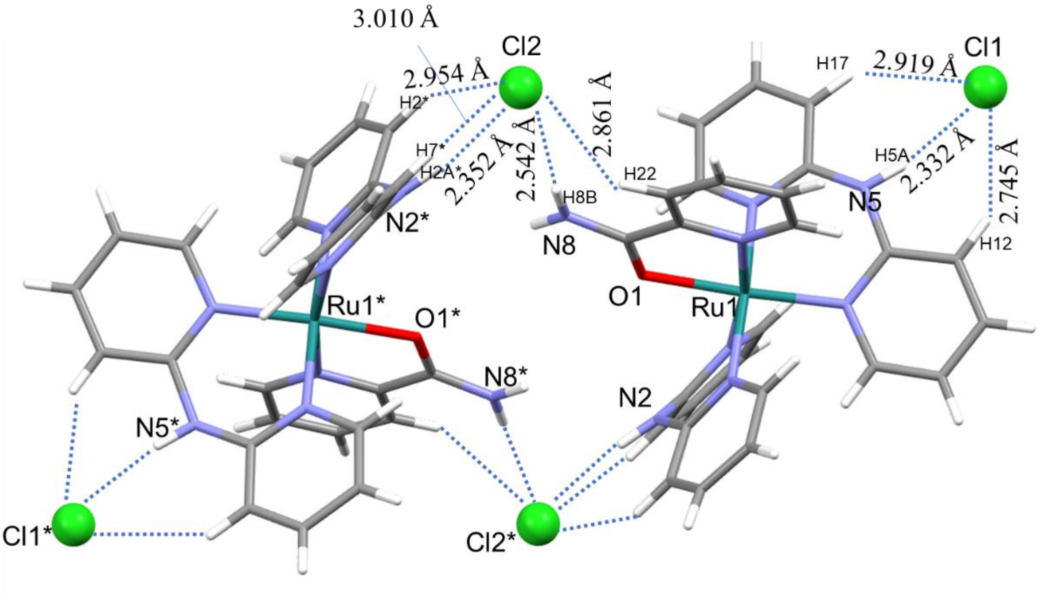

(b)

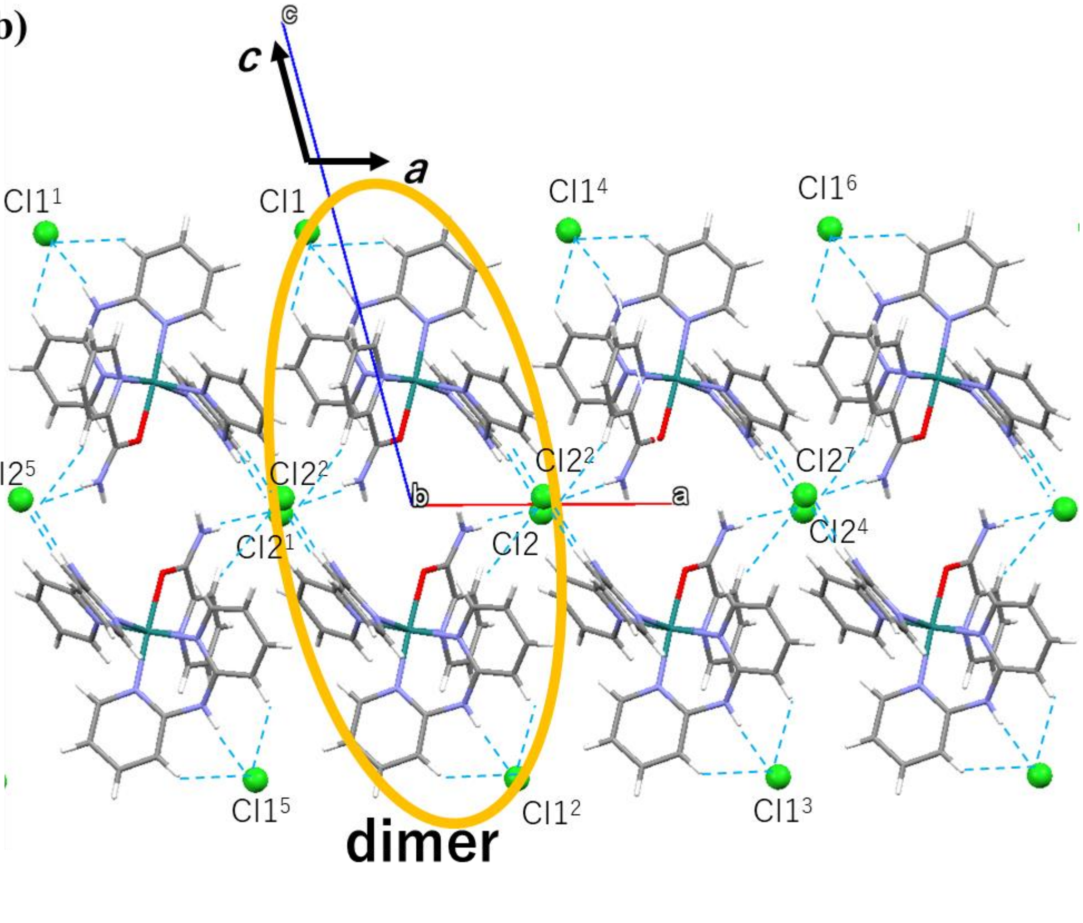

Fig. S8. 1D chain of dimer in crystal of **1**·Cl_2. (a) dimer unit and (b)1D chain of dimer. [(*) -x, -y, -z, (1) -1+x, y, z, (2) -x, -y, -z, (3) 1-x, -y, -z, (4) 1+x, y, z, (5) -1-x, -y, -z, (6) 2+x, -y, -z, (7) 2-x, -y, -x]

(a)

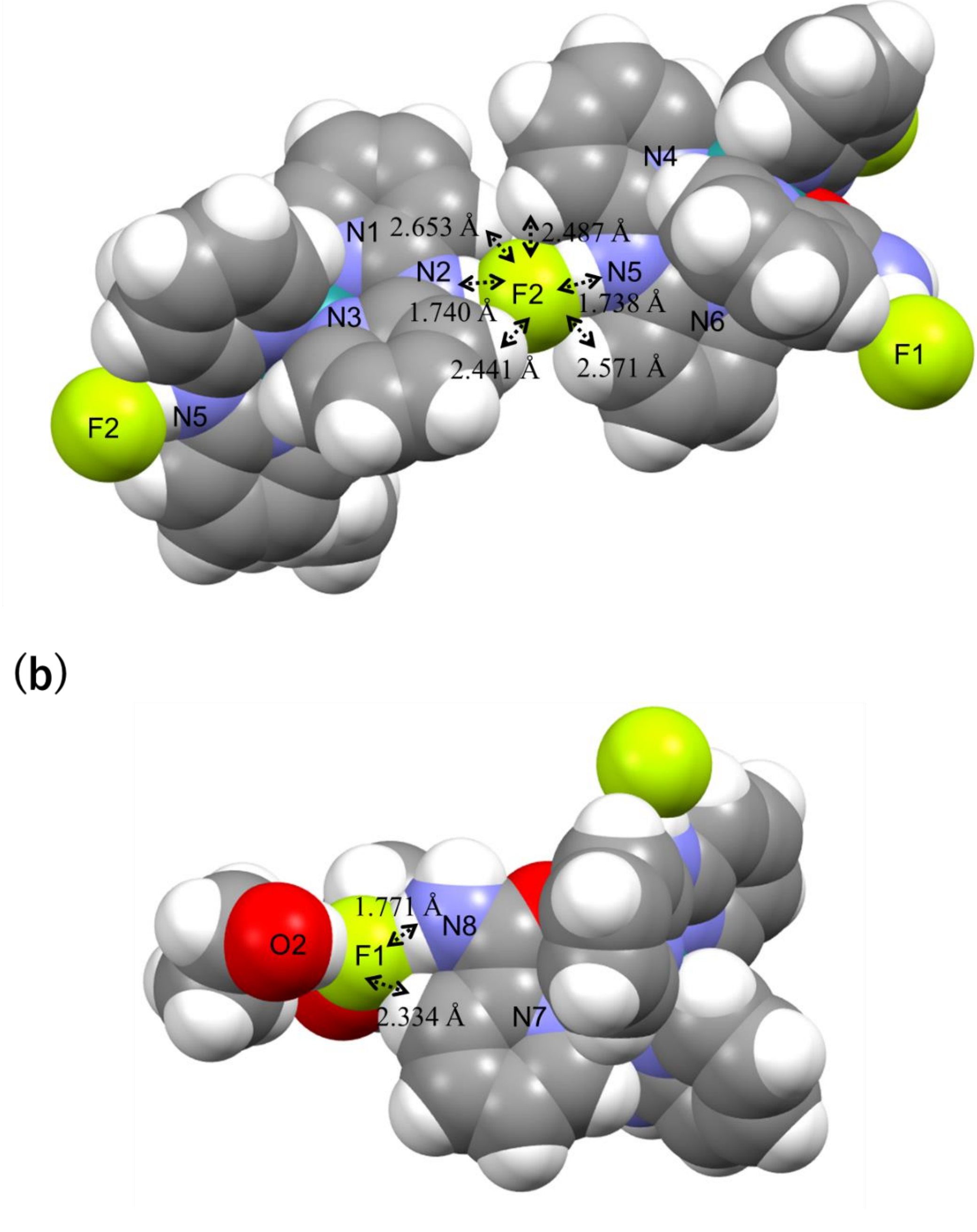

(b)

Fig. S9. Space Filling models of **1**·F_2·2EtOH.

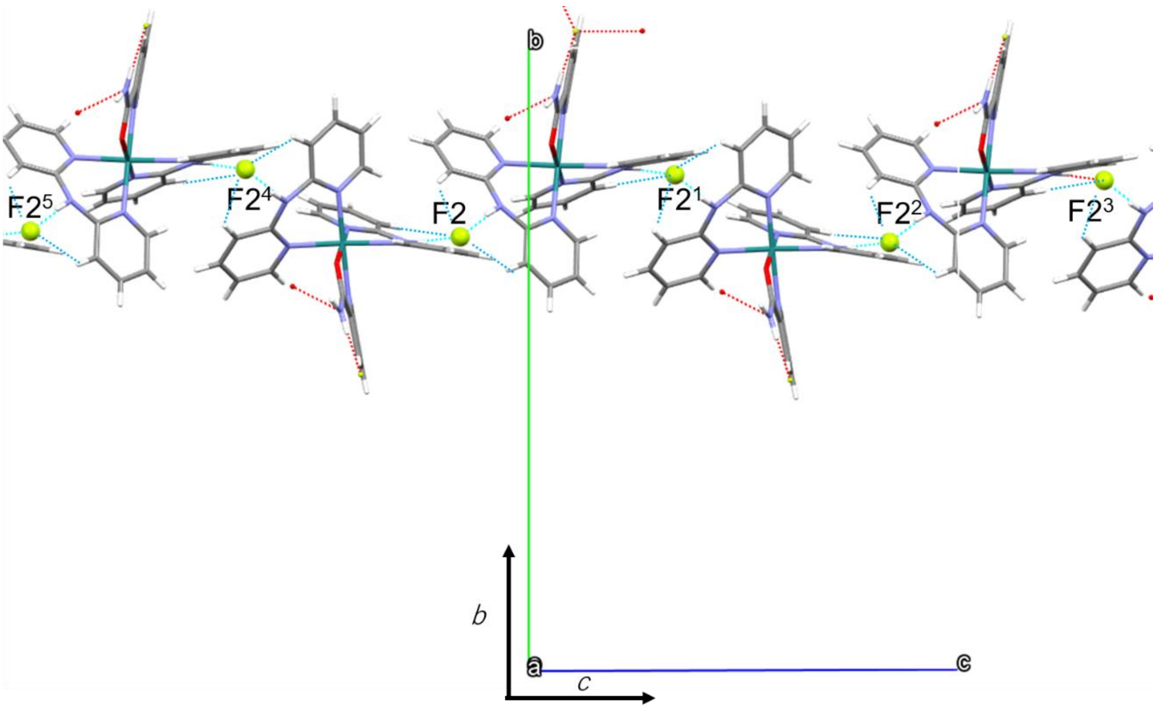

Fig. S10. 1D chain in crystal of **1**·F_2·2EtOH. [(1)1+x, 1.5-y, 1/2+z, (2)2+x, y, 1+z, (3) 3+x, 1.5-y, 1.5+y, (4) -1+x, 1.5-y, 1/2+z, (5) -1+x, y, -1+z]

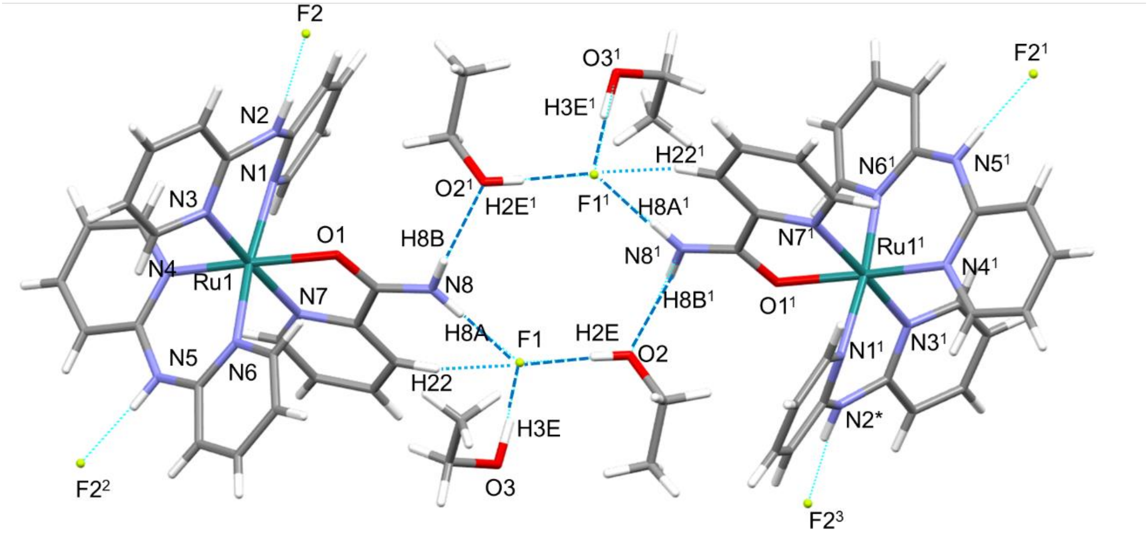

Fig. S11. Hydrogen bonding interaction between two amide group on two cationic complexes in crystal of **1**·F_2·2EtOH.
[(1)-x, 2-y, -z, (2) 1+x, 1.5-y, 1/2+z, (3) -x, 2-y, -z]

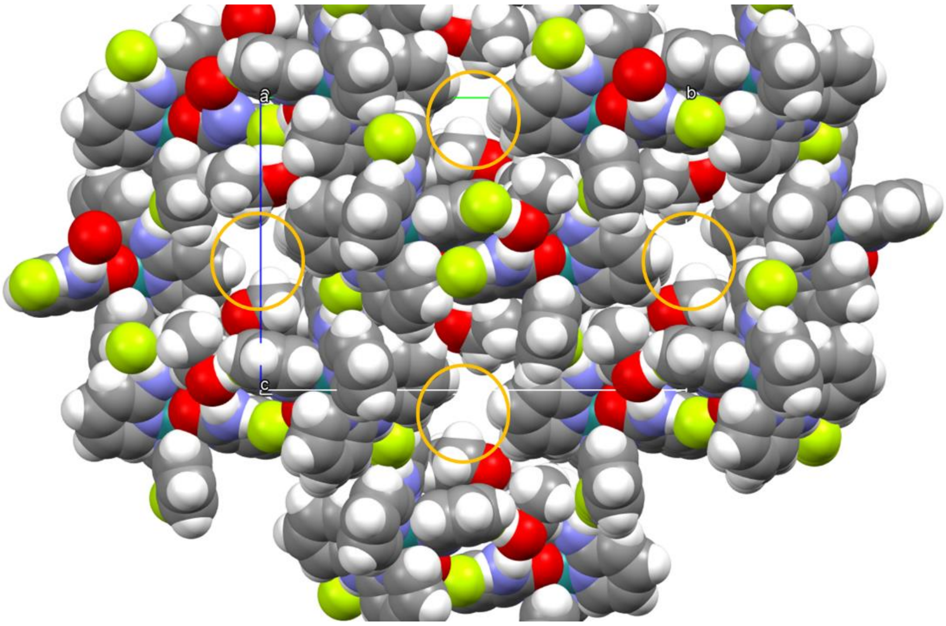

Fig. S12. Voids of crystal of **1**·F_2·2EtOH.

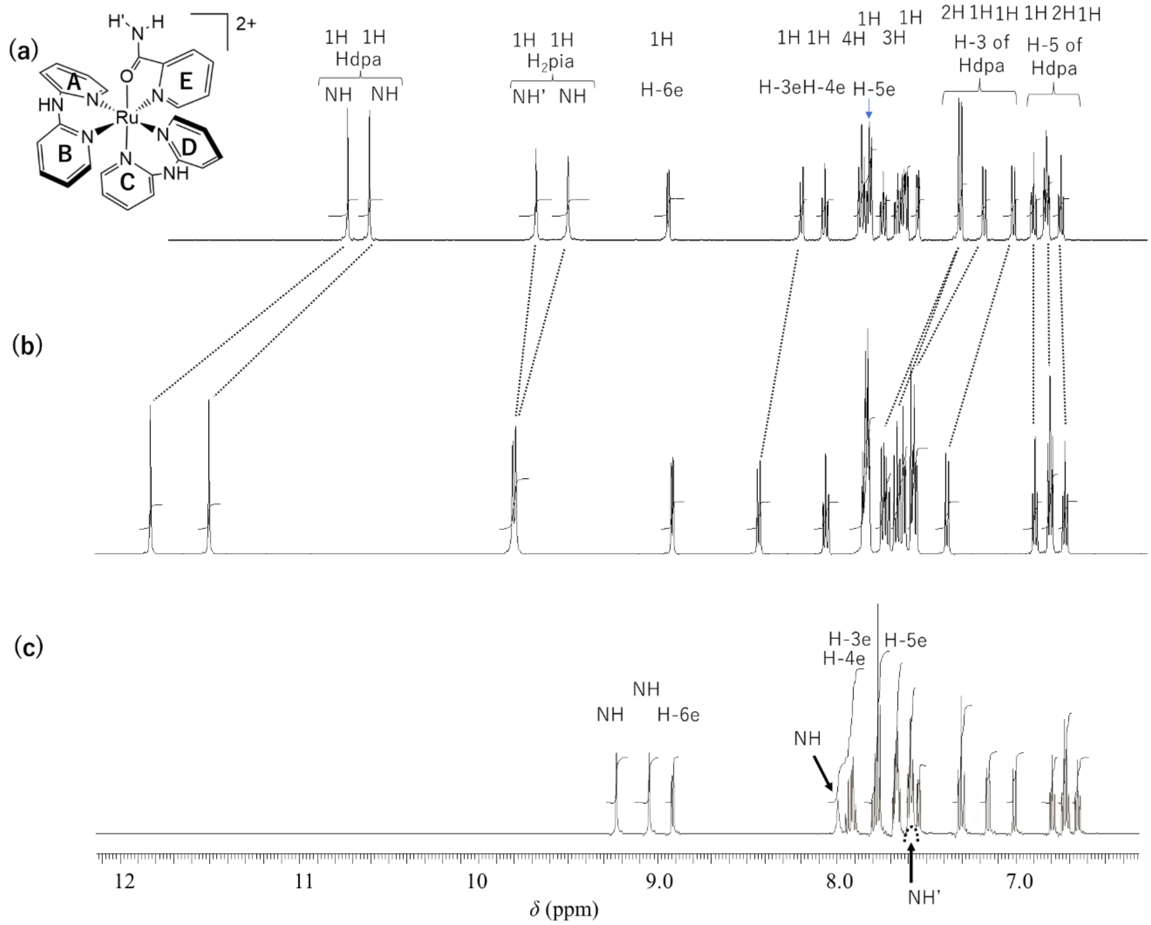

Fig. S13. ^{1}H NMR spectra (500 MHz, 298 K) of (a) **1**·$(OTf)_2$·H_2O in DMSO-d_6, (b) **1**·Cl_2·$2.5H_2O$ in DMSO-d_6, and (c) **1**·$(OTf)_2$·H_2O in CD_3CN.

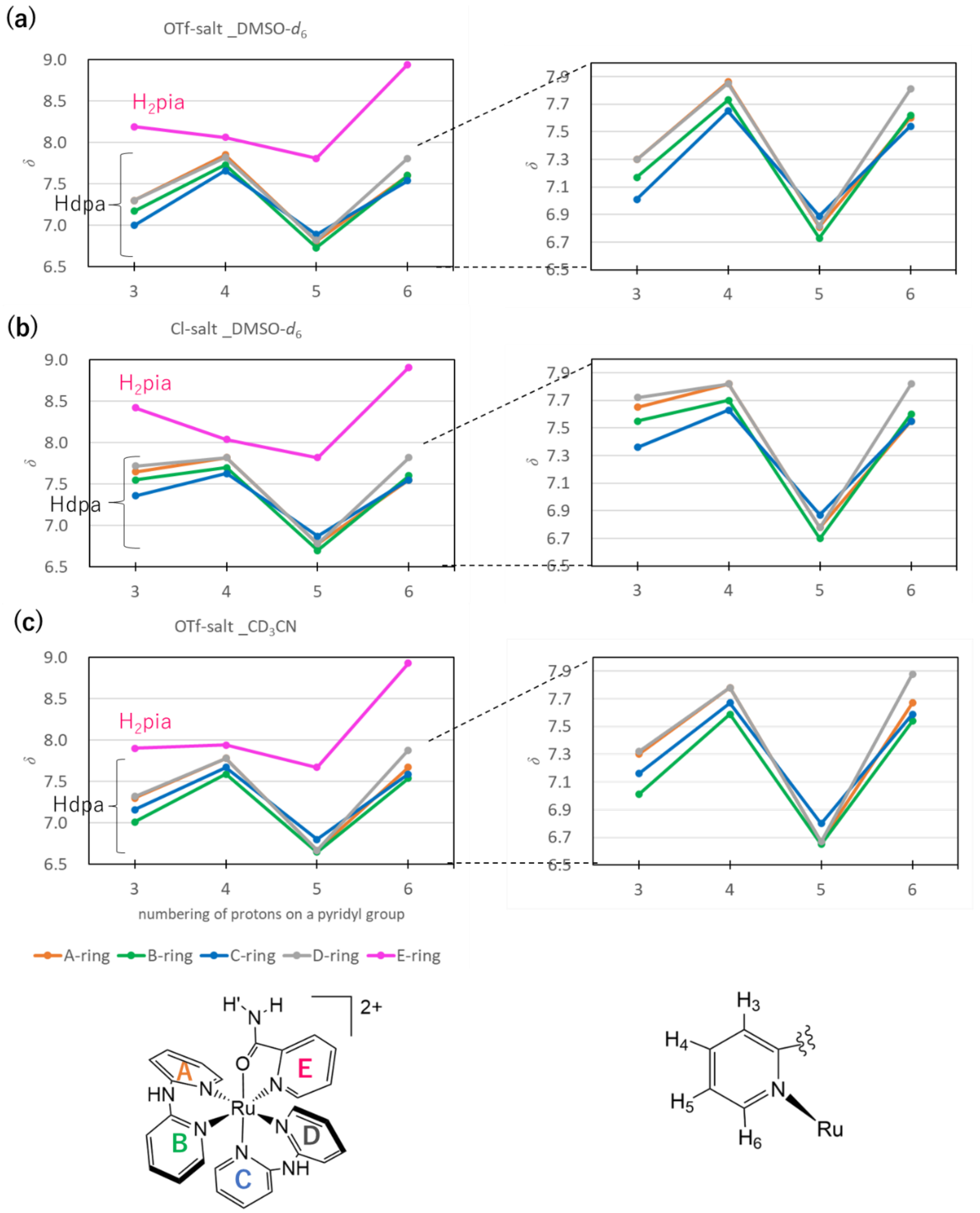

Fig. S14. Plots of the ^{1}H NMR chemical sifts of (a) **1**·$(OTf)_2$·H_2O in DMSO-d_6, (b) **1**·Cl_2·2.5H_2O in DMSO-d_6, and (c) **1**·$(OTf)_2$·H_2O in CD_3CN.

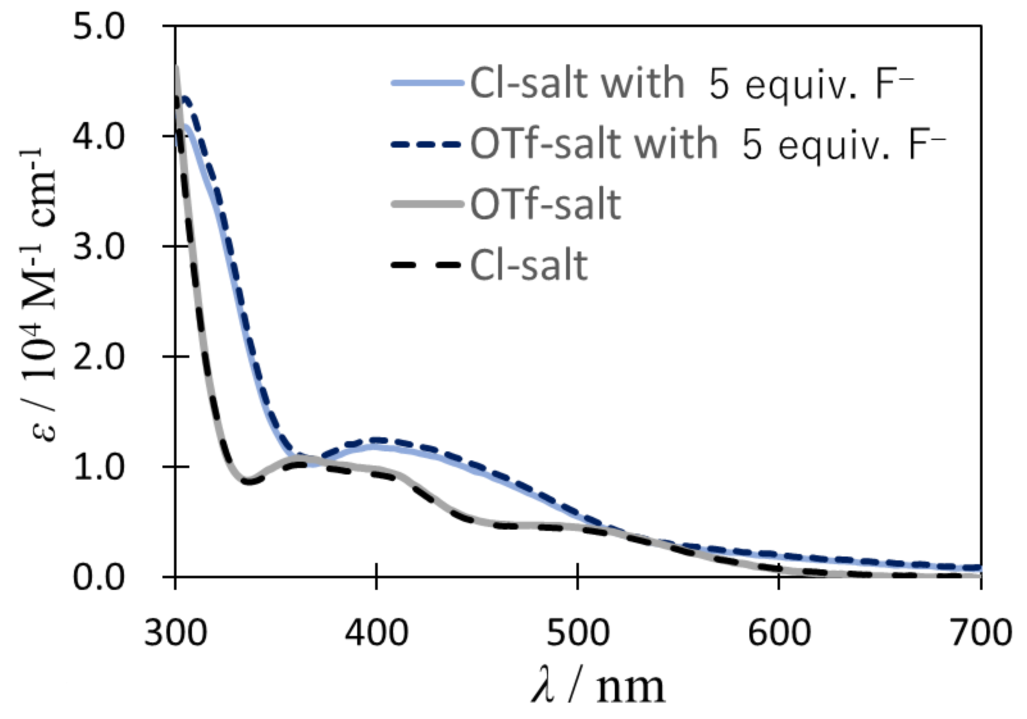

Fig. S15. Absorption spectra of **1**·$(OTf)_2$·H_2O (black bot line), **1**·Cl_2·$2.5H_2O$ (gray line), **1**·$(OTf)_2$·H_2O with 5 equivalents of F^- anions (blue bot line), and **1**·Cl_2·$2.5H_2O$ with 5 equivalents of F^- anions (pale blue line) in DMSO (2.0×10^{-5} M).

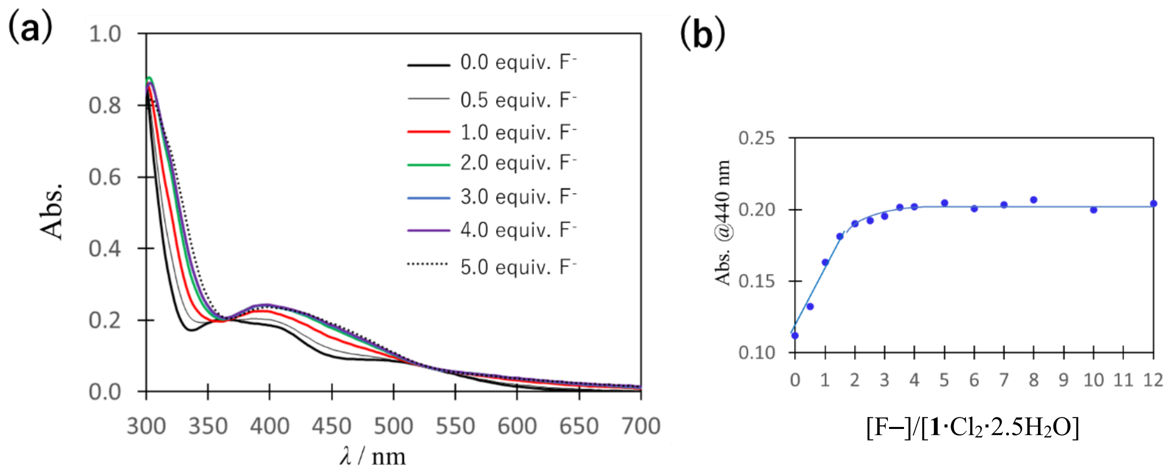

Fig. S16. (a) Family of absorption spectra taken over the course of the titration of a 2.0×10^{-5} M DMSO solution of **1**·Cl_2·$2.5H_2O$ with a standard solution of TBAF. (b) Titration profiles at 440 nm.

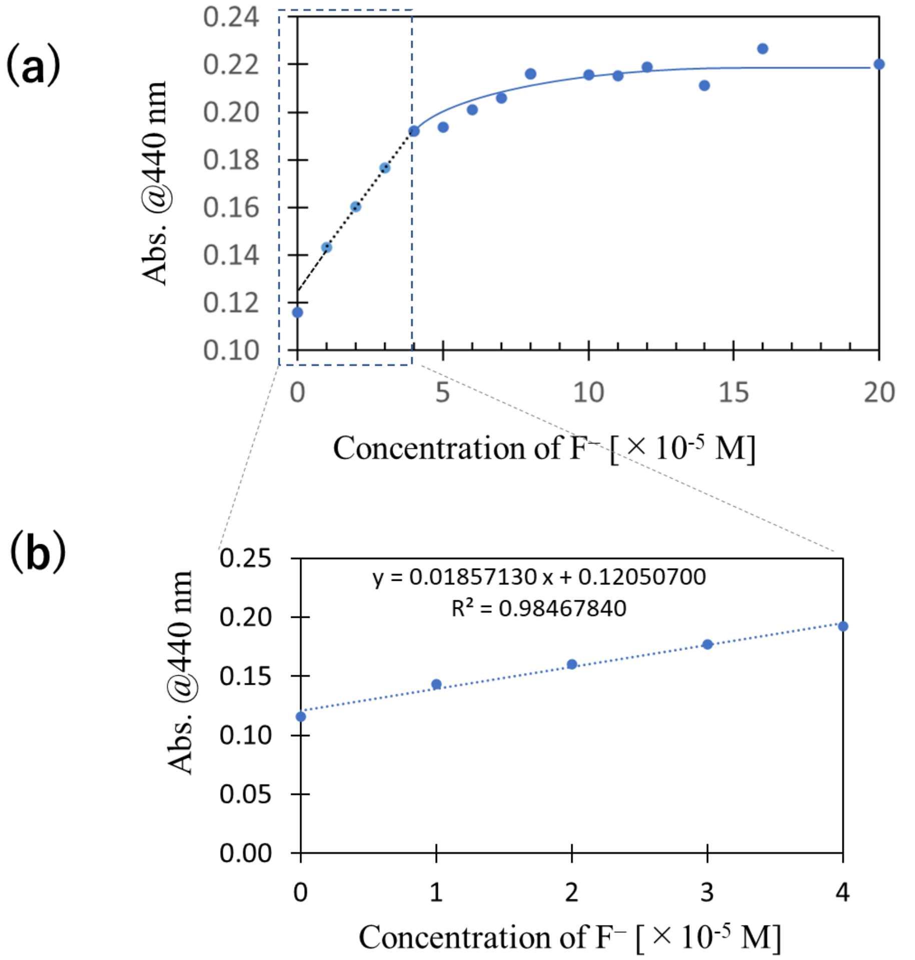

Fig. S17. Titration plot of absorbance of **1**·$(OTf)_2$·H_2O in DMSO (2.0 × 10^{-5} M) at 440 nm vs. concentration of F^- anion.

The limit of detection (LOD) was determined from the flowing equation: LOD = (3 × σ) / slope, where $\sigma = \{\Sigma(y_i - \tilde{y}_i)^2 / (n - 2)\}^{1/2}$ and 3is the factor at the 99% confidence level. [rel. 41,42] From the graph (x = 0 ~ 2.0 × 10^{-5} M), we get slope = (0.01857 / 10^{-5}), n = 5, and σ = 0.04229. Thus using the formula was get the LOD = 0.68 × 10^{-5}.

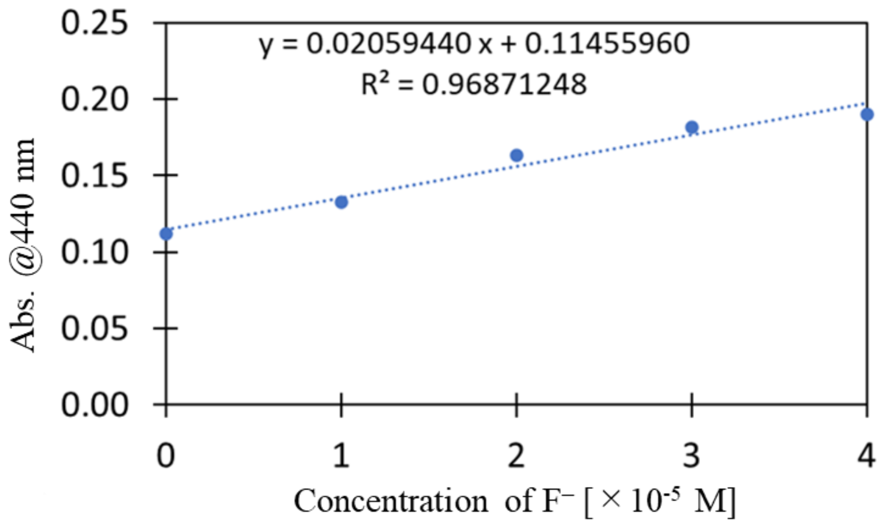

Fig. S18. Titration plot of absorbance of **1**·Cl_2·2.5H_2O in DMSO (2.0 × 10^{-5} M) at 440 nm vs. concentration of F^- anion.

The limit of detection (LOD) was determined from the flowing equation: LOD = (3 × σ) / slope, where $\sigma = \{\Sigma(y_i - \tilde{y}_i)^2 / (n - 2)\}^{1/2}$ and 3is the factor at the 99% confidence level. [ref. 41,42] From the graph (x = 0 ~ 2.0 × 10^{-5} M), we get slope = (0.02059 / 10^{-5}), n = 5, and σ = 0.06128. Thus using the formula was get the LOD = 0.89 × 10^{-5}.

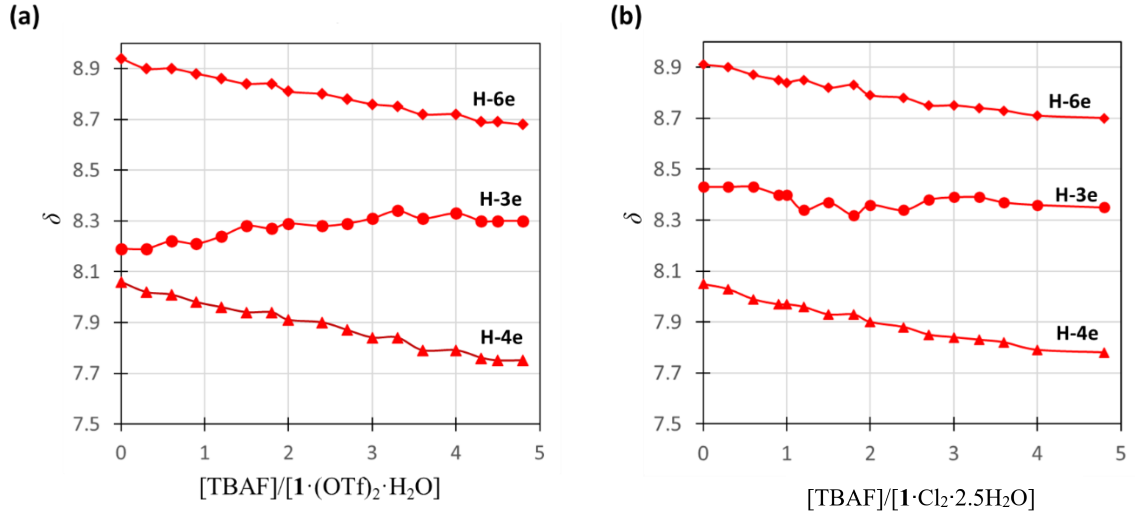

Fig. S19. Titration profiles; chemical shifts of H-3e, H-4e, and H-6e signals of H_2pia ligands in **1**·$(OTf)_2$·H_2O or **1**·Cl_2·2.5H_2O in DMSO-d_6 vs [TBAF]/[**1**·$(OTf)_2$·H_2O] or [TBAF]/[**1**·Cl_2·2.5H_2O], respectively.

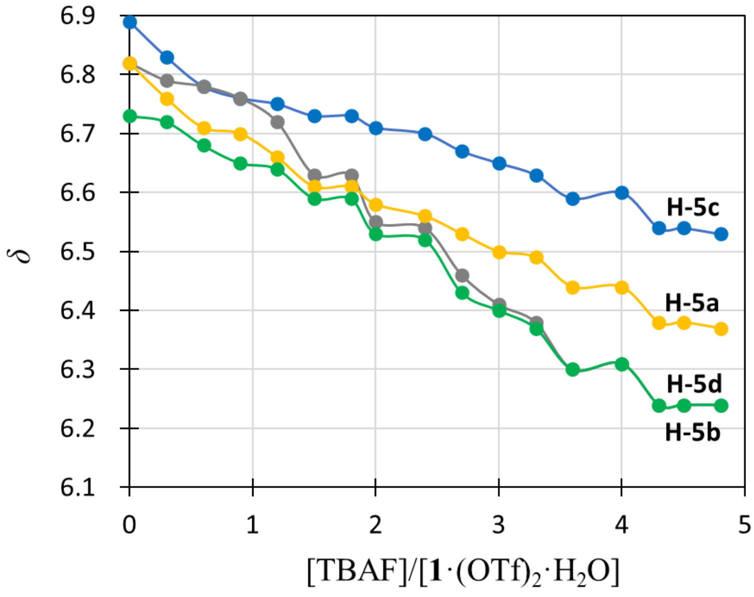

Fig. S20. Titration profiles; chemical shifts of H-5 signals of Hdpa ligands in **1**·$(OTf)_2$·H_2O in DMSO-d_6 vs [TBAF]/[**1**·$(OTf)_2$·H_2O].

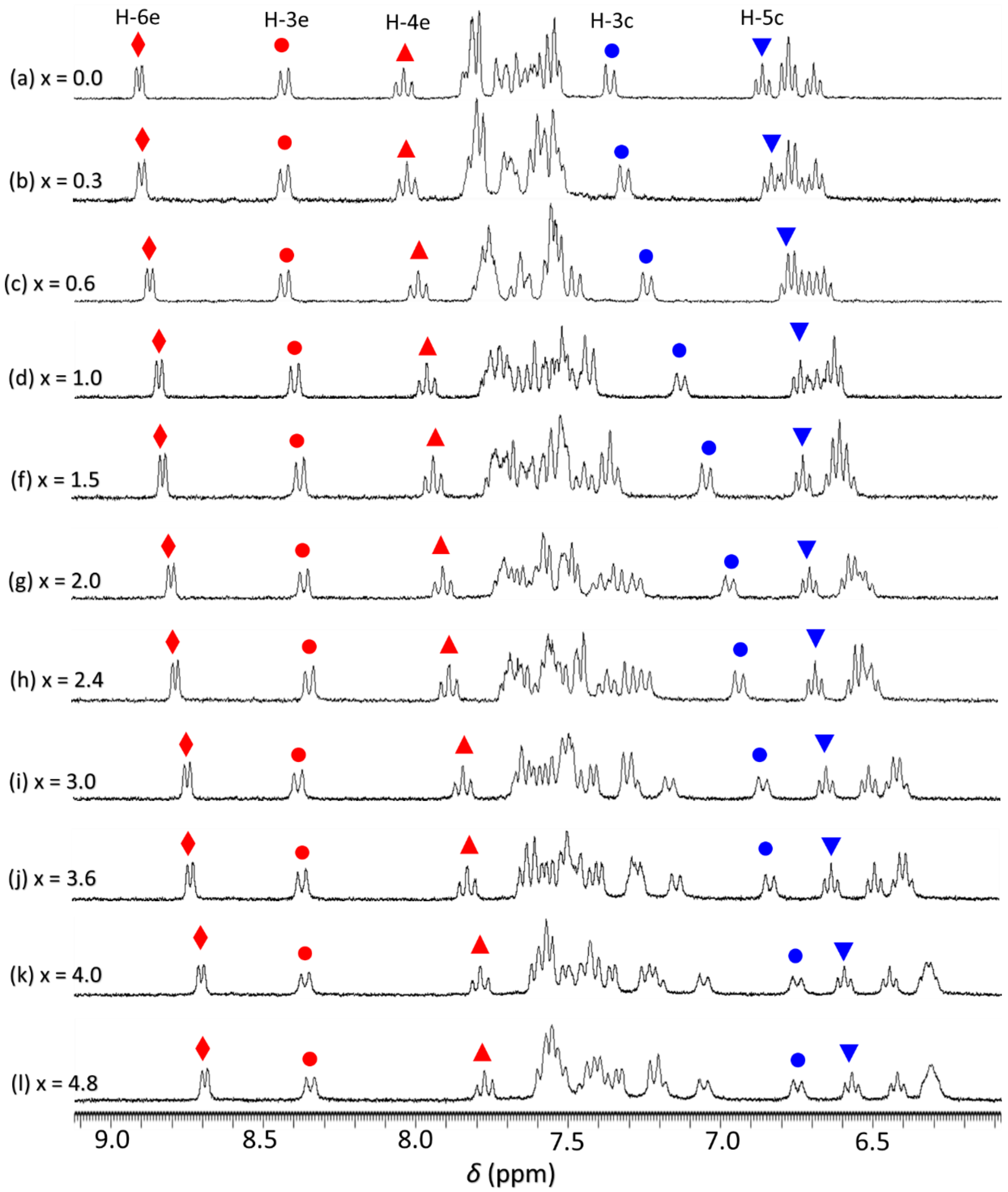

Fig. S21. ^{1}H NMR spectra taken over the course of a DMSO-d_6 solution of **1**·Cl_2·2.5H_2O (2.2×10^{-2} M) with TBAF. x = [TBAF]/[**1**·Cl_2·2.5H_2O]. (300 MHz NMR, 298 K)

	**1**$^{2+}$	**1**$\cdot F_3^-$
LUMO+4	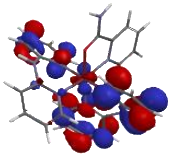 -6.29 eV	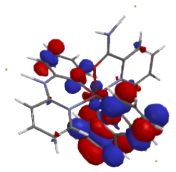 1.92 eV
LUMO+3	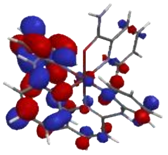 -6.53 eV	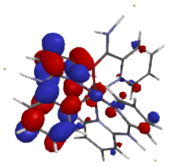 1.65 eV
LUMO+2	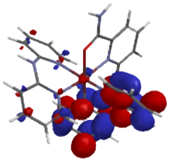 -6.58 eV	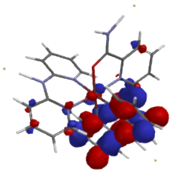 1.54 eV
LUMO+1	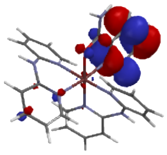 -6.71 eV	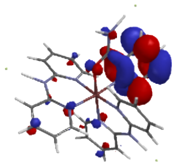 1.49 eV
LUMO	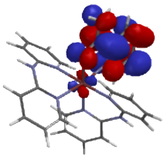 -7.62 eV	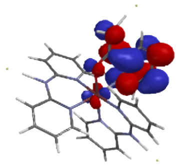 0.99 eV
HOMO	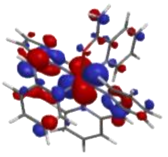 -10.78 eV	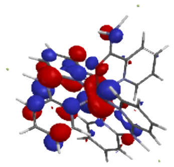 -2.02 eV
HOMO-1	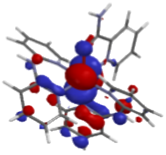 -10.85 eV	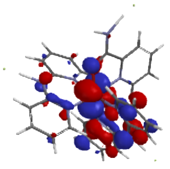 -2.1 eV
HOMO-2	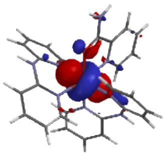 -11.16 eV	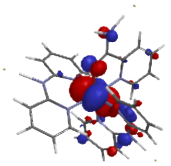 -2.44 eV

Fig. S22. Selected contour plots (isovalue 0.032 e/Å^3) of frontier orbitals and energy of **1**$^{2+}$ (left) and **1**$\cdot F_3^-$ (right) involved in the lower-lying transition.
